# Longitudinal effects of SARS-CoV-2 breakthrough infection on imprinting of neutralizing antibody responses

**DOI:** 10.1016/j.ebiom.2024.105438

**Published:** 2024-11-09

**Authors:** Sebastian Einhauser, Claudia Asam, Manuela Weps, Antonia Senninger, David Peterhoff, Stilla Bauernfeind, Benedikt Asbach, George William Carnell, Jonathan Luke Heeney, Monika Wytopil, André Fuchs, Helmut Messmann, Martina Prelog, Johannes Liese, Samuel D. Jeske, Ulrike Protzer, Michael Hoelscher, Christof Geldmacher, Klaus Überla, Philipp Steininger, Ralf Wagner, CoVaKo Study group, Helmut Messmann, Andre Fuchs, Alanna Ebigbo, Christoph Römmele, Maximilian Ullrich, Marie Freitag, Claudia Traidl-Hoffmann, Mehmet Goekkaya, Aline Metz, Corinna Holetschek, Avidan Neumann, Reinhard Hoffmann, Elisabeth Kling, Mihail Pruteanu, Thomas Wibmer, Susanne Rost, Klaus Überla, Philipp Steininger, Monika Wytopil, Stephanie Beileke, Sandra Müller-Schmucker, Klaus Korn, Tamara Hastreiter, Kirsten Fraedrich, Debora Obergfäll, Frank Neumann, Claudia Kuhn, Katja Günther, Elke Friedrich, Michael Hoelscher, Andreas Wieser, Christof Geldmacher, Christian Janke, Michael Plank, Jessica Guggenbühl, Christina Reinkemeyer, Ivan Noreña, Noemi Castelletti, Raquel Rubio Acero, M.I.M. Ahmed, Paulina Diepers, Tabea M. Eser, Anna Fuchs, Olga Baranov, Bernadette Bauer, Danni Wang, Ivana Paunovic, Ulrike Protzer, Samuel D. Jeske, Catharina Christa, Kathrin Tinnefeld, Martin Vu, Annika Willmann, Hedwig Roggendorf, Nina Körber, Tanja Bauer, Sabine Gleich, Ralf Wagner, Claudia Asam, Sebastian Einhauser, Manuela Weps, Antonia Senninger, George Carnell, Jonathan Luke Heeney, Antonia Ebner, Maria José de Schultz, Cedric Rajes, Aya Al Wafai, David Brenner, Laura Sicheneder, Melanie Berr, Anja Schütz, Stilla Bauernfeind, Andreas Hiergeist, André Gessner, Barbara Schmidt, Hans-Helmut Niller, Jürgen Wenzel, Daniela Biermeier, Benedikt Lampl, Ulrich Rothe, Ute Gleißner, Susanne Brückner, Michaela Treml, Holger Schedl, Beate Biermaier, Markus Achatz, Daniela Hierhammer, Johanna Englhardt, Werner Scheidl, Sivaji Jeyaraman, Barbara Schutt, Johannes Liese, Martina Prelog, Giovanni Almanzar, Valeria Schwägerl, Julia Bley, Tim Vogt, Kimia Kousha, Lars Ziegler, Astrid Stein, Franziska Förg, Johann Löw, Barbara Finkenberg, Dennis Pollak, Alexander Zamzow, Nicole Eberbach, Lara Balkie, Tanja Kretzschmann, Matthias Gehrig, Matthias Bandorf, Kilian Keck, Jan Allmanritter, Shahid Rafique, Mona Finster, Ingo Baumgart, Sabine Heumüller-Klug, Hans-Jürgen Koglin, Olaf Gefeller, Christine Gall, Annette B. Pfahlberg, Isabelle Kaiser, Jörg Scheidt, Johannes Drescher, Yannic Siebenhaar, Florian Wogenstein, Dirk Reinel, Beatrix Weber, Fabian Zarzitzky, Bernhard Liebl, Caroline Herr, Katharina Katz, Andreas Sing, Alexandra Dangel

**Affiliations:** a Institute of Medical Microbiology and Hygiene, Molecular Microbiology (Virology), University of Regensburg, Regensburg, Germany; b Institute of Clinical Microbiology and Hygiene, University Hospital Regensburg, Regensburg, Germany; c Department of Infection Prevention and Infectious Diseases, University Hospital Regensburg, Regensburg, Germany; d Laboratory of Viral Zoonotics, Department of Veterinary Medicine, University of Cambridge, Cambridge, United Kingdom; e DIOSynVax, Ltd., Cambridge, United Kingdom; f Internal Medicine III - Gastroenterology and Infectious Diseases, University Hospital of Augsburg, Augsburg, Germany; g Pediatric Rheumatology / Special Immunology, Department of Pediatrics, University Hospital Würzburg, Würzburg, Germany; h Pediatric Infectious Diseases, Department of Pediatrics, University Hospital Würzburg, Würzburg, Germany; i Institute of Virology, Technical University of Munich, TUM School of Medicine and Health, Munich, Germany; j Institute of Virology, Helmholtz Munich, Munich, Germany; k Division of Infectious Diseases and Tropical Medicine, University Hospital, Ludwig-Maximilians-Universität (LMU) Munich, Munich, Germany; l German Centre for Infection Research (DZIF), Partner Site Munich, Munich, Germany; m Institute of Clinical and Molecular Virology, Universitätsklinikum Erlangen, Friedrich-Alexander-Universität Erlangen-Nürnberg, Erlangen, Germany; n German Centre for Infection Research, Munich Partner Site, Germany

**Keywords:** SARS-CoV-2, COVID-19 breakthrough infection, Neutralization, Immune imprinting, Magnitude-breadth, Antigenic map, Machine learning

## Abstract

**Background:**

The impact of the infecting SARS-CoV-2 variant of concern (VOC) and the vaccination status was determined on the magnitude, breadth, and durability of the neutralizing antibody (nAb) profile in a longitudinal multicentre cohort study.

**Methods:**

173 vaccinated and 56 non-vaccinated individuals were enrolled after SARS-CoV-2 Alpha, Delta, or Omicron infection and visited four times within 6 months and nAbs were measured for D614G, Alpha, Delta, BA.1, BA.2, BA.5, BQ.1.1, XBB.1.5 and JN.1.

**Findings:**

Magnitude-breadth-analysis showed enhanced neutralization capacity in vaccinated individuals against multiple VOCs. Longitudinal analysis revealed sustained neutralization magnitude-breadth after antigenically distant Delta or Omicron breakthrough infection (BTI), with triple-vaccinated individuals showing significantly elevated titres and improved breadth. Antigenic mapping and antibody landscaping revealed initial boosting of vaccine-induced WT-specific responses after BTI, a shift in neutralization towards infecting VOCs at peak responses and an immune imprinted bias towards dominating WT immunity in the long-term. Despite that bias, machine-learning models confirmed a sustained shift of the immune-profiles following BTI.

**Interpretation:**

In summary, our longitudinal analysis revealed delayed and short lived nAb shifts towards the infecting VOC, but an immune imprinted bias towards long-term vaccine induced immunity after BTI.

**Funding:**

This work was funded by the Bavarian State Ministry of Science and the Arts for the CoVaKo study and the ForCovid project. The funders had no influence on the study design, data analysis or data interpretation.

**Research in context**

**Evidence before this study**

Numerous vaccines have been authorized to target the SARS-CoV-2 virus. However, the emergence of new immune escape variants poses an ongoing threat, leading to a significant rise in vaccine breakthrough infections. Various risk factors contributing to breakthrough infections, including declining immunity, age, and the specific vaccination or infection regimen, have been identified. Moreover, recent evidence strongly suggests the relevance of immune imprinting — a phenomenon previously observed in individuals infected with other viruses like influenza A — in understanding vaccine efficacy and SARS-CoV-2 immunity. However, most studies on immune imprinting suffer from limited participant numbers or lack long-term follow-up data.

The critical question that remains is: How does immune imprinting influence the neutralizing antibody profiles against SARS-CoV-2 following repeated exposures to diverse antigens? Understanding the potential impact of immune imprinting is paramount for assessing existing immunity against SARS-CoV-2 and developing effective vaccines.

**Added value of this study**

This study endeavours to shed light on the ramifications of immune imprinting on neutralizing antibody profiles following breakthrough infections, utilizing a cohort of 229 participants tracked over time. Notably, it stands as the pioneering effort in scrutinizing the longitudinal impact of vaccine-mediated immune imprinting on the evolution of neutralization profiles using advanced techniques such as antigenic mapping and antibody landscaping. Moreover, we introduce the application of Kaplan-Meier-based analysis, well-established in the HIV domain, to explore the magnitude and breadth of neutralization against various SARS-CoV-2 variants of concern (VOC).

Our findings indicate that breakthrough infections with Delta or Omicron variants, which are antigenically more distant to the vaccine, tend to elicit a robust, enduring, and broad neutralizing antibody (nAb) response. The evolution of neutralizing antibody profiles over time varies significantly depending on vaccination history and the specific VOC responsible for the infection. Notably, the longitudinal analysis reveals an initial augmentation of the vaccine-primed nAb response upon infection, followed by a progressive expansion of neutralization capacity towards the infecting SARS-CoV-2 variant. Long-term observation reveals a subsequent contraction and inclination towards dominant wild-type (WT) immunity post-breakthrough infection.

Furthermore, this study marks a significant milestone as it demonstrates, for the first time, the efficacy of AI/machine learning models in accurately identifying the SARS-CoV-2 VOC that caused the breakthrough infection based on the post-infection neutralizing antibody profile. This underscores a sustained shift in long-term immunity following breakthrough infections, notwithstanding the imprinting bias inherent in the immune response.

**Implications of all the available evidence**

Immune imprinting poses a persistent challenge in both vaccine development and the assessment of SARS-CoV-2 immunity. Studies utilizing antibody depletion and longitudinal BCR sequencing have compellingly demonstrated the existence of B-cell specificities imprinted by initial immunizations. Subsequent exposures with different antigens serve to reinforce and broaden these specificities, yet don't elicit novel specificities, finally resulting in a recurring bias towards the initial antigen. However, emerging evidence suggests that prolonged exposure to the same novel antigen may eventually override immune imprinting. Furthermore, machine learning techniques indicate that even a single encounter with a different antigen can lead to a lasting reshaping of the elicited neutralizing antibody profile. Moreover, repeated immunizations and specific booster antigens influence both the magnitude and sustainability of the neutralizing antibody response. Collectively, these findings offer crucial insights for designing novel vaccines aimed at achieving broad and ideally sterilizing immunity against Coronaviruses.

## Introduction

The appearance of new SARS-CoV-2 immune escape variants remained an ongoing threat and resulted in high numbers of vaccine breakthrough infections (BTI) over the last 2 years.^1^

Multiple risk factors for a BTI^2^ - such as time after vaccination, age, previous disease, vaccine type, and circulating VOC - have been identified in various studies,^3^^,^^4^ including a waning of vaccine-elicited antibodies against SARS-CoV-2 infection over time.^5^^,^^6^ Furthermore, the rapid emergence and global spread of Alpha, Delta, and Omicron VOCs impressively demonstrated that neither prior infection nor vaccination efficiently protected from infection with new VOCs.^7^

Regarding heterologous vaccine booster immunizations,^5^ combinations of vaccination and infection (“hybrid immunity”),^8^ especially breakthrough infection (BTI), or repeated infection with different VOCs,^9^ questions were raised whether immune imprinting^10^ might be relevant in cases of preexisting immunity. This phenomenon, also known for other respiratory viruses such as the influenza virus, might limit broadening of immune responses following repeated exposure to variants of the same antigen.^11^, ^12^, ^13^, ^14^

Since serum neutralization capacity was identified as a key correlate of protection from COVID-19,^15^, ^16^, ^17^ the impact of prior vaccination on neutralizing antibody responses (nAb) following SARS-CoV-2 infection with different VOCs is of high importance.^18^, ^19^, ^20^

Accordingly, based on a cohort of 229 individuals with documented vaccination, infection, and clinical history, this longitudinal study aims to elucidate the interplay of prior vaccination and BTI with different SARS-CoV-2 VOCs on the kinetic, magnitude, breadth, and durability of neutralizing antibody responses compared to infection in unvaccinated individuals. Using antigenic mapping and neutralization landscaping as advanced bioinformatic tools, this study will also provide insight into the potential impact of vaccine-induced immune imprinting on neutralization capacity following BTI.

## Methods

### Sampling

Blood samples of individuals from a prospective longitudinal multicentre cohort study (CoVaKo) of acute SARS-CoV-2 BTIs and non-BTIs were analysed. Study centres were the respective University Hospitals in Erlangen, Regensburg, Augsburg, Würzburg, and Munich (TUM and LMU), all located in Bavaria, Germany. The study design is described in Prelog et al.^21^

In brief, samples of 56 non-vaccinated and 173 vaccinated individuals with a newly diagnosed SARS-CoV-2 infection were collected between April 2021 and April 2022 for Alpha (n = 32), Delta (n = 134) infections and between January and August 2022 for Omicron infections (n = 63). Individuals with vaccination received at least two vaccinations with wildtype-based mRNA vaccines (BNT162b2, Comirnaty, BioNTech/Pfizer and mRNA-1273, Spikevax, Moderna (n = 155)) or vector-based vaccines (AZD1222, Vaxzevria, AstraZeneca and JNJ-78436735, Jcovden; Janssen-Cilag/Johnson&Johnson (n = 8)) or a combination of vector and mRNA-based vaccines (n = 10) (Table 1, Supplementary Table S1).

**Table 1:** Demographic characteristics of the study cohort.

Vaccination Status	Vaccinated	Unvaccinated	Vaccinated	Unvaccinated	Vaccinated	Unvaccinated	All	All	All	Total	Vaccinated	Unvaccinated
Infection with	Alpha	Alpha	Delta	Delta	Omicron	Omicron	Alpha	Delta	Omicron	Total	Total	Total
n	24	8	94	40	55	8	32	134	63	229	173	56
Age (median)	40	35.5	38.5	31.5	38	30	39.5	37.5	34	38	39	31.5
Age (IQR)	34–46.3	30.8–42.8	29–50.8	27–46.5	28.5–45.5	29–37.5	32.8–46.3	28–49	29–45.5	29–48	29–49	28–46.5
Female n	18	6	53	25	29	4	24	78	33	135	100	35
Female %	75	75	56.4	62.5	52.7	50	75	58.2	52.4	59	57.8	62.5
Days after vax (median)	76	–	95	–	108	–	76	95	108	95	95	–
Days after vax (IQR)	61.8–81.8	–	56.3–134.5	–	85–140	–	61.8–87.8	56.3–134.5	83.5–140	65–132.6	65–133	–
Current smoking n	5	1	15	6	5	1	6	21	6	33	25	8
Smoking %	20.8	12.5	16	15	9.1	12.5	18.8	15.7	9.5	14.4	14.5	14.3

Including the numbers of participants for the respective subgroups, as well as age, sex, smoking status, vaccination status and days between last vaccination and study inclusion (days after vax, “–” means undeterminable due to no vaccination received). Abbreviations: number (n), interquartile range (IQR).

The first study visit took part within 14 days after the first positive SARS-CoV-2 RT-qPCR test (day 1, visit V1) and included a VOC-specific PCR for determining the SARS-CoV-2 VOC. Follow-up visits were carried out on day 8 ± 1 (V2), day 15 ± 1 (V3), day 22 ± 2 (V4) and between 4 and 6 months (V5) after the initial visit, respectively. As V3 was an optional visit according to the study protocol, which was only carried out in a minority of individuals, this study visit was not included in this analysis. The participant sex data was self-reported.

### Pseudotype neutralization assay

The capacity of sera to neutralize different SARS-CoV-2 variants was determined using a lentiviral pseudotype assay, as described previously.^22^

In brief, an inoculum of 2.5∗10ˆ5 rlu/384-well of lentiviral particles expressing luciferase and pseudotyped with SARS-CoV-2 spike protein was neutralized with a 2-fold serum dilution series for 1 h. Luciferase activity was determined 48 h post-infection of HEK293T-ACE2 + -cells using BrightGlo (Promega Corp, Madison, WI, USA). The 50% inhibitory dilution (ID50) of the sera was calculated using GraphPad Prism 8 (San Diego, CA, USA) after normalizing to noninfected and infected cells and curve fitting with the algorithm “log (inhibitor) vs. normalized response”. Neutralizing antibody titres were determined against the variants WT/D614G, Alpha (B.1.1.7), Delta (B.1.617.2), Omicron BA.1, BA.2, BA.5 and BQ.1.1, XBB1.5 and JN.1 (for V5).

### Reagent validation

The HEK293T-ACE2+ cell line was kindly provided by Stephan Pöhlmann, it was sourced from the DSMZ (Cat. No.: ACC-635, RRID: CVCL_0063) and validated in his Lab. ACE-2 expression was validated by Western Blotting. Cells were regularly tested for Mycoplasma infection by PCR (MycoSPY Master Mix, Biontex, M020-050).

### Magnitude-breadth analysis

As both, magnitude (nAb titre) and breadth (percentage of VOCs neutralized) of serum neutralization are important criteria for the evaluation of humoral immunity, we utilized the concept of magnitude-breadth curves, as described early for the analysis of humoral HIV immunity.^23^, ^24^, ^25^ In brief, for each serum and time point, a Kaplan–Meier style curve was calculated using the neutralization data against all tested variants. Curves were calculated with “50% neutralization of one variant” defined as the “event of interest” and the “time-to-event” representing the 50% inhibitory concentration (IC50) for that variant. Thus, yielding a curve representing the magnitude of the immune response on the x-axis and the breadth, represented by the percentage of variants neutralized, on the y-axis. Magnitude-breadth curves for each group were calculated as the sum of individual curves divided by the number of individuals in the group. In addition, areas under the curve (AUC) were calculated for each magnitude breath curve, which facilitates the comparison of neutralization magnitude-breadth results between the different groups and visits.

### Antigenic cartography

Antigenic maps and antibody landscapes were calculated in R^26^ as previously described by others.^27^, ^28^, ^29^ In brief, antigenic cartography quantifies and visualizes neutralization data. It uses a two-dimensional antigenic map to represent the difference in log2 titres between antiserum S and antigens A and B. Modified multidimensional scaling arranges the points on the map to match the target distances from the neutralization data. The resulting map shows the antigenic distance, with distances between antigens and antisera inversely related to log2 titres. Antigenic maps were computed using the Racmacs package.^30^ Maps were constructed with 1000 optimizations, setting the minimum column basis parameter to “none”. Map bootstrap was performed by randomly resampling the dataset with replacement 1000 times, calculating new maps with those samples and reoptimizing each map 100 times, as further specified in the racmacs package reference. Finally, a blob representing one standard deviation of the antigen position was calculated from the resulting bootstrap positions.

Antibody landscapes were generated by adding a third dimension to the antigenic map represented by the geometric mean titres to each variant and subsequently fitting a cone-shaped object. Landscapes were calculated using the ablandscapes package by Sam Wilks.^31^ Further packages used were: tidyverse, meantiter, r3js, htmlwidgets, webshot 2, grid, gridExtra, and patchwork.

### Regression analysis

After explorative Spearman correlation, generalized additive regression in R was used to jointly investigate the association of quantitative nAb levels against the BTI variant (IC50) with time after vaccination until BTI and breakthrough variant among vaccinated study participants. Separate models were calculated for the different visits. Smoothing terms were calculated using a thin plate regression spline basis, with automatic selection of the effective degrees of freedom. We illustrated the model results by inspecting the estimated (non-linear) associations and visualizing the predicted antibody levels over the range of observed times after the last vaccination until alpha, delta, and omicron infected, respectively. The models were estimated using the mgcv-package in R.^32^

### Adjustment to study centre effects and varying days of the visit

To adjust the neutralization data for study centre effects and varying visit days, a Tobit model was selected as the most suitable approach due to the upper and lower censoring of the neutralization data. Biologically, different directions and strengths of visit day effects are expected at different time points (e.g. increasing titres over time at visit 1 vs. waning titres at visit 5). Therefore, according to the principle of parsimony, separate (simpler) models were calculated for each visit opposed to one large but complex model accounting for those effects. To avoid overadjustment in the later visits, the day of the visit was scaled to the mean day of the current visit for each visit.

To ensure robustness, we chose a more stringent model using the larger groups of vaccinated and unvaccinated individuals, rather than the smaller groups also stratified by infection or breakthrough variant. This approach minimizes the risk of fitting bias due to single outliers in the smaller groups, yet it implies the risk of non-adjustment for individual effects in those smaller groups. For study centre effects, centre 5 (University Hospital Regensburg), which provided the most participants, was used as the baseline. Thus, all models were calculated with neutralization titres dependent on the neutralized virus variant, the number of days between the first positive PCR test and the current visit, vaccination status, study centres, and interaction effects between the visit day and vaccination status. Finally, all neutralization values were corrected for study centre effects, visit day effects, and the interaction effects of visit day and vaccination status, specific to each visit. Combined variance accounted for by the models was calculated by correlation of predicted values of all models combined, with measured values.

Finally, adjustment of neutralization values was performed utilizing the tobit-models’ effects as linear effects and applying post-adjustment censoring to the adjusted values to still comply with the experimental upper and lower bound of 1 and 2561. Cases with missing data were excluded from initial modelling, but if possible, adjusted using available data, else they were excluded.

### Machine learning models

Random forest models with 500 trees as well as neural networks were utilized to predict the infecting variant (Alpha, Delta, Omicron) of either vaccinated or unvaccinated individuals from their neutralization titres (IC50) against all tested VOCs. Thus, utilizing models with the titres against D614G, Alpha, Delta, BA1, BA2 and BA5 as features and virus (Alpha, Delta, Omicron) as a target on data filtered by vaccination status and visit. Data was split in half into training and testing data by random sampling of the single groups. To avoid learning bias from different group sizes we used a method inspired by bootstrapping with monte-carlo simulation. In brief, the amount of learning data was increased by random but group stratified resampling from the available training datasets to gain a total of 999 equally fractioned training datasets. To avoid overlearning from the resampling process, additional noise was introduced in all resampled values while keeping the overall neutralization profile. Single values were randomly drawn from a generated normal distribution of 50 values with a mean of the original value and a standard deviation of 10% of the original value. As a compromise of avoiding bias and improving accuracy, a brute-force approach was used: 100 models were calculated on the generated training data set and checked for predictive accuracy using the respective test dataset. After keeping the best model, novel datasets were generated as described above and utilized to calculate 100 new models. This process was looped 100 times finally reporting the mean metrics of all 100 models calculated on the different datasets. Separate models were calculated for the vaccinated and unvaccinated subgroups as well as for the different visits. For neural networks, hyperparameter selection (3–5 neurons in the first, 0 to 3 neurons in the second hidden layer) was performed by calculating 10 models of each possible configuration, using the dataset for vaccinated individuals at visit 4, the visit with the assumed highest effect and the lowest chance of bias. The simplest configuration resulting in the best mean accuracy was chosen. Finally, shallow neural networks with 5 neurons in one hidden layer, using a maximum of 109 gradient steps for adjusting the weights of the network and a threshold of 0.1 for the partial derivatives of the error function to stop learning. Networks were trained for 100 independent datasets, generated as described above, finally reporting the mean accuracy of all models. Models were calculated in R using the randomForest and neuralnet packages.

### Statistical analysis

For intergroup comparison, we used Kruskal–Wallis tests combined with post-hoc Dunn's-corrected multiple comparison tests, while we used Friedman tests with Bonferroni-corrected post-hoc Wilcoxon testing for longitudinal/paired comparisons (e.g. visits). Two vs. three times vaccinated groups were compared using a Mann–Whitney U test. Individuals with Adenovirus/Vector vaccination were compared to individuals with mRNA vaccination using Mann-Whitney-U tests. The proportion of current smokers, defined by self-reported cigarette smoking, and sex distribution for individual groups was evaluated using Fisher's exact tests. Age differences between groups as well as the days between vaccination and infection were assessed using the aforementioned Kruskal-Wallis-tests. Differences between magnitude-breadth curves were assessed by log-rank-tests, while differences between the AUCs were assessed by Kruskal–Wallis tests combined with post-hoc Dunn's-corrected multiple comparison tests. Finally, due to unknown demographics and total numbers of the infected population with or without vaccination at the time of sampling it was impossible to judge the cohort's representativeness or perform valid sample size calculations, which should be considered when interpreting the study results and is further discussed later.

### Multivariate analysis

For multivariate analysis we utilized PERMANOVA models with post hoc pairwise comparison, due to the non-normality of censored neutralization data. As some possible influencing variables like number of vaccines, vaccine type/vector vaccine or days_since_vax were non applicable for unvaccinated individuals, and missing values are unsupported by the method, two models were calculated. Modelling neutralization distances at the four visits as a function of age, gender, smoking status, virus to be neutralized, infection variant for the unvaccinated individuals, adding in the aforementioned vaccination specific variables for the vaccinated individuals. To adjust post-hoc comparison, we utilized a tobit-model to retrieve residuals of neutralization as a function of visit, age, gender, smoking status, virus to be neutralized. Additionally, for vaccinated individuals including number of vaccines, vaccine type/vector vaccine and days_since_vax. Here, infection variant was not included as it ought to be post-hoc analysed. Afterwards, we performed pairwise PERMANOVA on the distance matrix of residuals.

### Software

Statistical analysis was performed using SPSS Statistics (version 29.0.0.0 (241), IBM Corp. Released 2021. IBM SPSS Statistics for Windows, Version 28.0. Armonk, NY, USA), GraphPad Prism 8 for Windows (version 8.0.1, GraphPad Software, San Diego, CA, USA) or R (version 4.2.3, R core team, Vienna, Austria) in RStudio (2023.12.0 Build 369, RStudio Team, Boston MA, USA).

### Ethics

Written informed consent was provided by all study participants, the study was approved by the Ethics Committee of the Friedrich-Alexander-University Erlangen-Nürnberg, Germany (vote 46_21 B) and adopted by the local ethics committees of all other study centers. The study complies with the 1964 Declaration of Helsinki and its later amendments. The Clinical Trials registration was DRKS00024739.

### Role of funders

This work was funded by the Bavarian State Ministry of Science and the Arts for the CoVaKo study and the ForCovid project. The funder had no influence on the study design, data analysis or data interpretation.

## Results

### Descriptive analysis of the study cohort

In total, 229 individuals (173 BTIs and 56 non-BTIs) were included (Table 1). The majority of the two-times and the three-times vaccinated individuals received the BNT162b2mRNA (BioNTech/Pfizer) vaccine (n = 104, 83.2% and n = 36, 75.0%, respectively) (Supplementary Table S1). The vaccinated group comprised of individuals with a median age of 39 years (IQR 29–49), 57.8% (n = 100) being women and 14.5% (n = 25) current smokers whilst the unvaccinated group included individuals with a median age of 32 years (IQR 28–47), a female share of 62.5% (n = 35) and 14.3% (n = 8) current smokers. Comparing variant-stratified vaccinated and unvaccinated groups, no significant differences in age (p = 0.443), sex (p = 0.481), or smoking status (p = 0.670) composition could be found. The median interval in days from the last vaccination was 76 ± 32, 95 ± 55, and 108 ± 46 days for Alpha, Delta, and Omicron BTIs, respectively. Comparing those intervals, we found significant differences between Alpha and Omicron BTIs (p = 0.002). Previous infection was excluded by N-antibody measurements, health authority data and self-reporting as described in Prelog et al.^21^

### Adjustment for follow up time and study centre effects

**Supplementary Figure S1:**
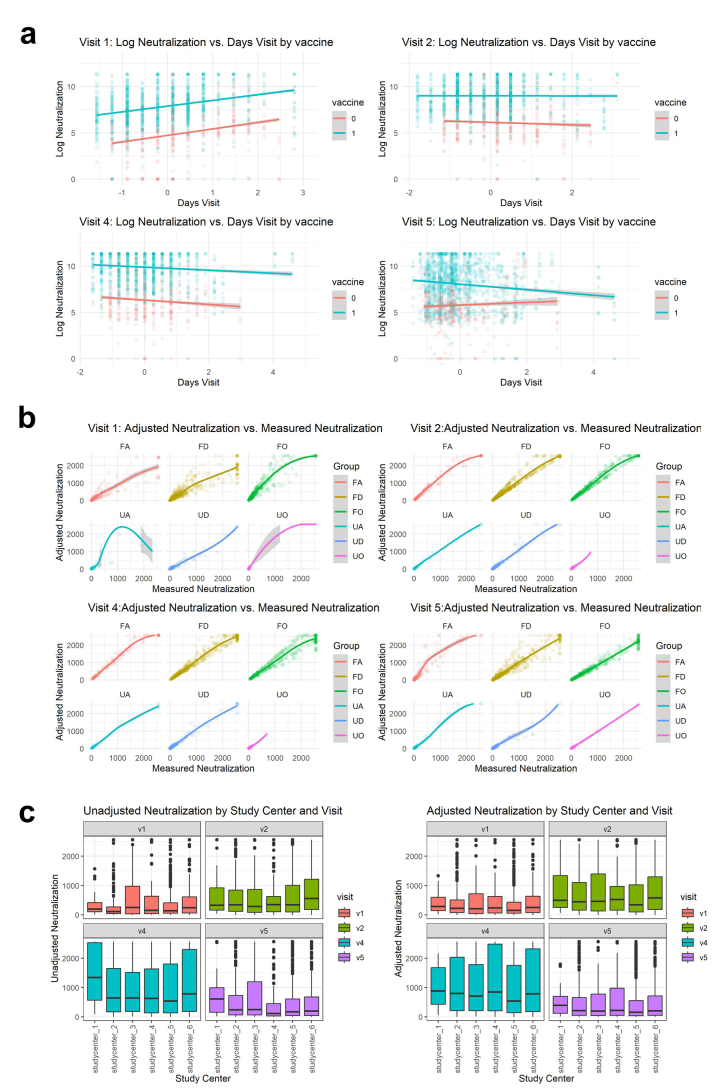
**Adjustment for follow up time and study centre effects**. Tobit models for every visit were utilized to adjust the neutralization data for follow up time (scaled to mean) and study centre effects with centre 5 as reference. **a** Visualization of assumed linear follow-up time effects by vaccination status (0 = no vaccination n = 56, 1 = vaccinated n = 171) and visit. **b** Visualization of the adjustment for each visit and within the different groups analysed later (FA = Vaccinated Alpha BTI n = 24, FD = Vaccinated Delta BTI n = 92, FO = Vaccinated Omicron BTI n = 55, UA = Unvaccinated Alpha Infection n = 8, UD = Unvaccinated Delta Infection n = 40, UO = Unvaccinated Omicron infection n = 8). Shown are adjusted values on the y axis and unadjusted values on the X axis with loess regression to visualize trends. **c** Visualization of study centre stratified nAb titres and study centre adjustment, shown are median titres for each visit by study centre. The left panel shows unadjusted values, the right panel the adjusted values.

As this study was comprised of individuals sampled in multiple study centres at varying time points for each visit, bias/effects from both, study centres or follow up time could not be excluded. On top, biologically, different effects (e.g. raising titres with time after the initial infection, waning titres at the long term visit) were to be expected from follow up time for the different visits. Thus, all neutralization data was adjusted for those effects utilizing four tobit models, one for each visit. Combining the predictions of the single models for the whole dataset, overall variance accounted for by the models was 0.69. The models identified several significant influences of all, study centres (2 and 4 at visit 1; 2,3 and 4 at visit 2; 2 and 4 at visit 4; 1,3 and 4 at visit 5), follow up time (visit 1 and visit 4) and interaction of vaccination status and follow up time at visit 5, justifying the adjustment. Effect strength and direction were, as biologically expected, varying at the different visits, justifying the splitting of the models to the different visits (Supplementary Figure S1). However, those models could, because of smaller group sizes, not adjust for possible additional group specific effects, thus a remaining group specific bias from study centre and follow-up time cannot fully be ruled out.

### Neutralization assays of alpha/delta/omicron-infected groups

**Fig. 1:**
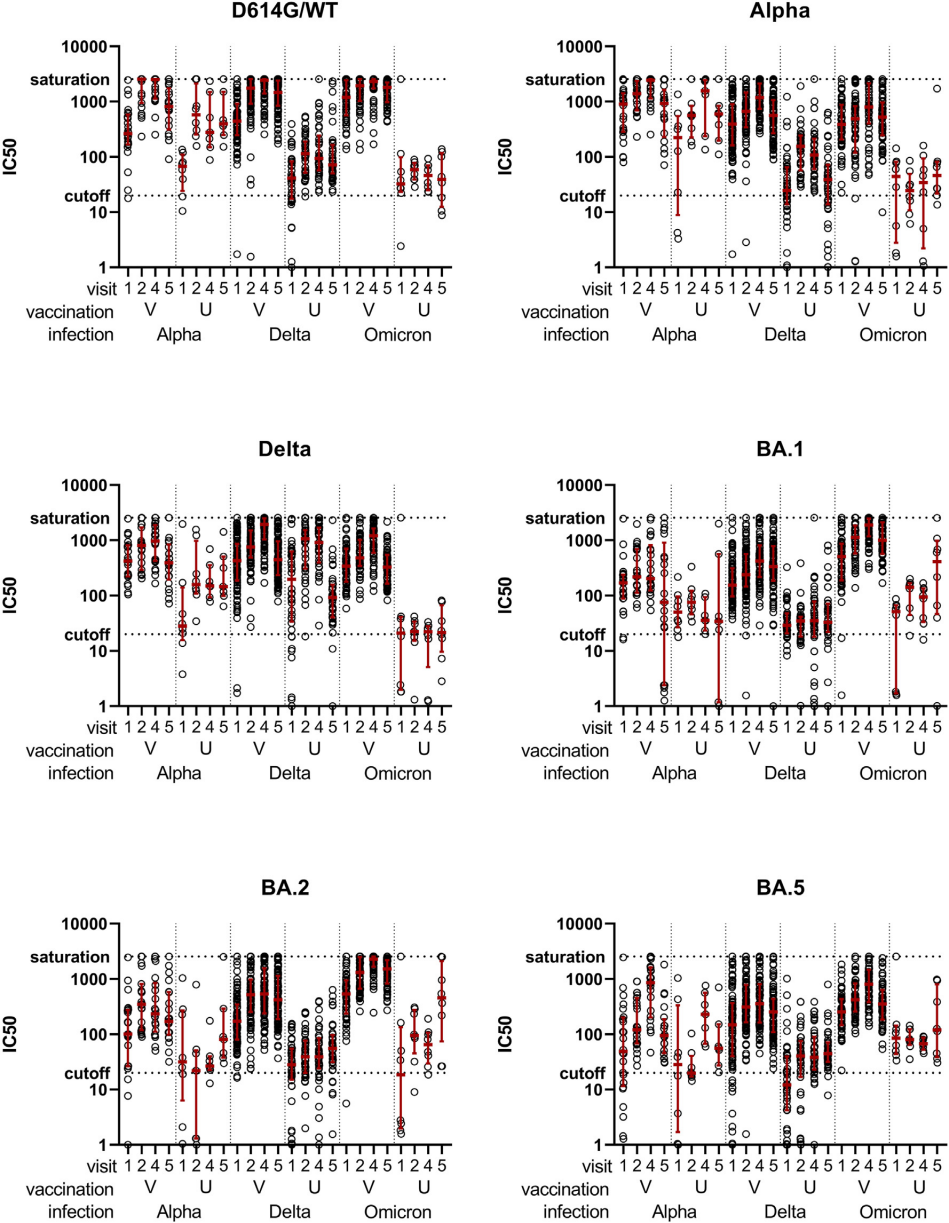
**Neutralization assays against different SARS-CoV-2 variants for vaccinated and unvaccinated Alpha, Delta and Omicron infected study groups**. Neutralizing antibody responses of vaccinated (V, n = 171) and unvaccinated (U, n = 56) individuals at visit 1, 2, 4 and 5 after infection with Alpha (A, n = 32), Delta (D, n = 132) or Omicron (O, n = 63) (as indicated on the x-axis). Shown are the dilution factors for 50% Inhibitory concentration (IC50) for WT/D614G, Alpha, Delta, Omicron BA.1, Omicron BA.2 and Omicron BA.5 spike, as indicated by the individual graph titles. The assay cut-off (IC50 = 20) as well as saturation (IC50 = 2560) are indicated by dashed lines. IC50s were measured in three replicates. Median and interquartile ranges are indicated by the red horizontal line and whiskers.

**Supplementary Figure S2:**
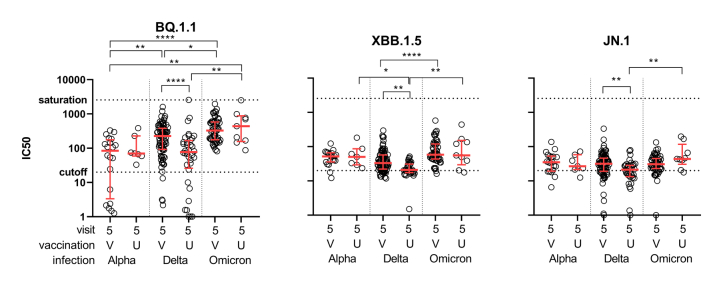
**Neutralization assays against the SARS-CoV-2 variant BQ.1.1 for vaccinated and unvaccinated Alpha, Delta and Omicron infected study groups at visit 5**. Antibody responses of vaccinated (V, n = 171) and unvaccinated (U, n = 56) individuals at visit 5 after infection with Alpha, Delta or Omicron (as indicated on the x-axis). Serum dilution factors needed to reach 50% inhibitory concentration (IC50) for Omicron BQ.1.1. The assay cut-off (IC50 = 20) as well as saturation (IC50 = 2560) are indicated by dashed lines. IC50 were determined by three replicates. Median and interquartile ranges are indicated in blue. Differences between vaccinated and unvaccinated were analysed by Mann-Whitney test, statistically significant differences are indicated by p values (∗∗ p < 0.005). [Mann-Whitney-U] Differences between infection variant groups were analysed by Kruskal-Wallis tests with post-hoc Dunn’s corrected multiple comparisons tests, statistically significant differences are indicated by p values (∗ p < 0.05,∗∗ p < 0.005, ∗∗∗ p< 0.001, ∗∗∗∗ p < 0.0001)[Kruskal-Wallis with post-hoc Dunn’s].

The samples of vaccinated and unvaccinated participants of the Alpha-, Delta- and Omicron-infected groups were analysed for their neutralizing antibodies against the WT/D614G and the VOCs Alpha, Delta, Omicron BA.1, BA.2, and BA.5 at four timepoints (V1, V2, V4, V5; see Fig. 1), and additionally against the VOCs BQ1.1, XBB.1.5 and JN.1 at V5 (Supplementary Figure S2).

In general, for infections with the SARS-CoV-2 Delta and Omicron VOC, a higher median neutralization capacity was found for vaccinated individuals compared to the unvaccinated groups at all visits, in the variants with minor antigenic distance against the vaccine antigen (e.g. D614G, Alpha, Delta). Nevertheless, a high nAb titre was found for unvaccinated individuals tested against their infecting variant. Noteworthy, Omicron infection in unvaccinated individuals resulted in overall low nAbs against all tested PV variants except for the last visit, where omicron-specific nAbs started to increase. Consequently, individuals with Omicron BTI did benefit most from prior vaccination regarding their nAb response against variants tested here with less antigenic distance to the vaccine antigen than BQ1.1, where overall nAb titres started to decline correspondingly.

Additionally, sera at V5 (140 d, IQR 133–150 d after infection) were analysed for their neutralization capacity against the Omicron variant BQ.1.1, XBB.1.5 and JN.1 (Supplementary Figure S2). Differences were analysed by Kruskal–Wallis tests with post-hoc Dunn's tests. The highest BQ.1.1 nAb titres were found in individuals with Omicron infection history, whilst median IC50s were comparable in vaccinated and unvaccinated individuals. BQ1.1 nAb titres in the Omicron infected were significantly higher than those determined in the Alpha and Delta infected individuals, regardless of vaccination status, respectively (p-values denoted in the figure). NAb titres were lowest after infection and BTI with the alpha VOC. Only in the Delta-infected group, vaccinated individuals showed higher nAb titres against BQ.1.1. in comparison to unvaccinated individuals (p = 0.0001)[ Dunn's]. Moving to the more recent variants XBB.1.5. and JN.1 a similar pattern was revealed, though with notably lower overall response revealing a loss of differences between the vaccinated and unvaccinated individuals with omicron infection history. Regarding vaccinated individuals: while for BQ.1.1 a significantly increased titre was found for omicron BTI compared to Delta BTI (p < 0.05) and Alpha BTI (p < 0.001), XBB.1.5 neutralization reached significance only for Delta BTI (p < 0.001)[ Dunn's]. Regarding JN.1 neutralization significance was lost for both Alpha and Delta BTI.

**Supplementary Figure S3:**
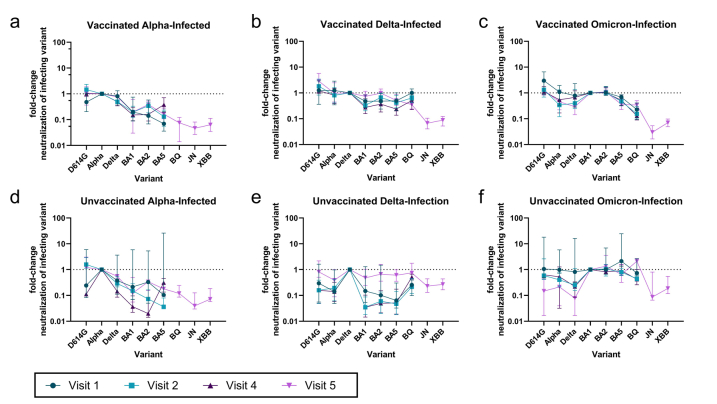
**Estimates of fold-change compared to the homologous variant**. Comparison of median fold differences for all measured SARS-CoV-2 variants relative to the titres against the variant of infection: Alpha panel **a** (vaccinated, n = 24) and **d** (unvaccinated, n = 8), Delta panel **b** (vaccinated, n = 92) and **e** (unvaccinated, n = 40), Omicron panel **c** (vaccinated, n = 55) and **f** (unvaccinated, n = 8). Visits 1, 2, 4 and 5 are shown simultaneous in each panel. Points show the median estimate for the fold-change difference while lines show the interquartile range for the estimate.

Furthermore, we determined the variant-specific neutralization profiles normalized to the infecting variant for each group (Supplementary Figure S3). Distinct neutralization profiles were found for Alpha, Delta, or Omicron infected individuals peaking for the infecting VOC, respectively. The vaccinated groups revealed an additional maximum for WT/D614G corresponding to the vaccine-encoded antigen (Supplementary Figure S3 a–c).

### Effects of booster vaccination, adenoviral-vector vaccination and time between vaccination and BTI

**Supplementary Figure S4:**
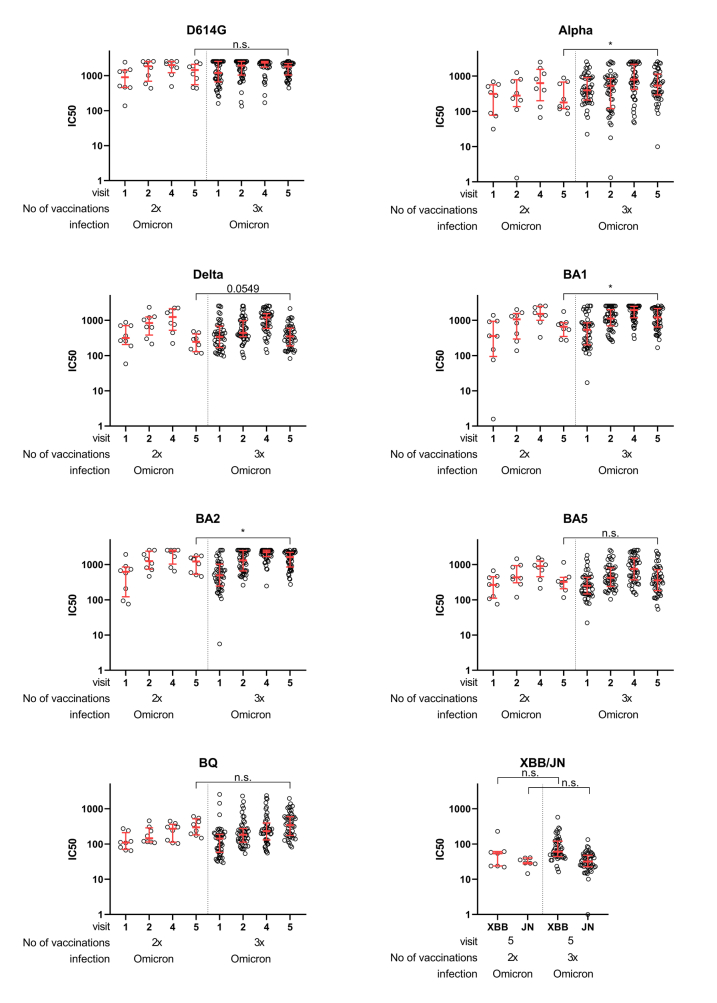
**Comparison of neutralization assays against different SARS-CoV-2 variants for double- and triple-vaccinated Omicron groups**. Antibody responses of double vaccinated (2V, n = 47) and triple vaccinated (3V, n = 8) individuals at visit 1, 2, 4 and 5 after infection with Alpha, Delta or Omicron (as indicated on the x-axis). Shown are the dilution factors for 50 % Inhibitory concentration (IC50) for WT/D614G, Alpha, Delta, Omicron BA.1, Omicron BA.2 and Omicron BA.5 spike, as indicated by the individual graph titles. The assay cut-off (IC50 = 20) as well as saturation (IC50 = 2560) are indicated by dashed lines. IC50s were determined in triplicates. Median and interquartile ranges are indicated in blue. Statistically significant differences are indicated by p values (∗ p < 0.05, ∗∗ p < 0.005).[Mann-Whitney-U].

An important covariate to be considered is the number of vaccinations. Therefore, we utilized our vaccinated Omicron group, the only one where individuals received either 2 or 3 vaccinations (Supplementary Table S1). Noteworthy, whereas no significant differences in neutralization capacity could be found for all variants during V1 – V4, the triple-vaccinated individuals revealed (significantly) higher IC50 titres for the VOCs Alpha (p < 0.05), Delta (p = 0.055), BA.1 (p < 0.05) and BA.2 (p < 0.05)[Mann–Whitney] at V5 compared to the double-vaccinated group (Supplementary Figure S4).

**Supplementary Figure S5:**
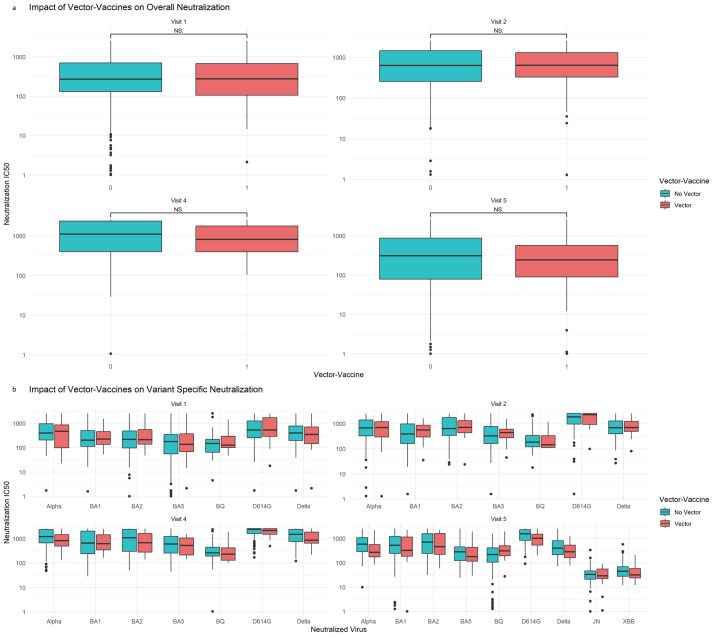
**Comparison of neutralization in adenoviral vector- or mRNA vaccinated individuals**. **a** Shown are pooled nAb titres against all tested variants (D614G, Alpha, Delta, BA1, BA2, BA5) in either individuals who received an adenoviral vaccination at any point (n = 18) or in individuals who only received mRNA vaccines (n = 153), for the different visits. Statistically significant differences are indicated by p values (∗ p < 0.05, ∗∗ p < 0.005)[Mann-Whitney-U] **b** Shown are variant specific nAb titres at four different visits in either individuals who received an adenoviral vaccination at any point (n = 18) or in individuals who only received mRNA vaccines. Statistically significant differences are indicated by p values (∗ p < 0.05, ∗∗ p < 0.005). [Mann-Whitney-U].

We further analysed the impact of adenoviral vector-based vaccines in comparison to vast majority of participants who received only mRNA based vaccines. Due to the heterogeneity of vaccination regimens (see Supplementary Table S1) in combination with low numbers of participants, all participants who received any vector-based vaccine (Janssen or Astra-Zeneca) at any point in their vaccination history (first, second, third or multiple) were pooled as the “vector-vaccinated group” (n = 18). To evaluate on general effects, neutralization titres against all variants were pooled as overall neutralization. Here, no significant differences between individuals in the vector-vaccinated group and individuals without any vector vaccination were found at any visit (Supplementary Figure S5a). Regarding variant and visit specific effects (Supplementary Figure S5b) we found significantly increased titres in the vector-vaccinated group at visit 5 against D614G and Alpha (p = 0.042 & p = 0.048) [Mann–Whitney].

**Supplementary Figure S6:**
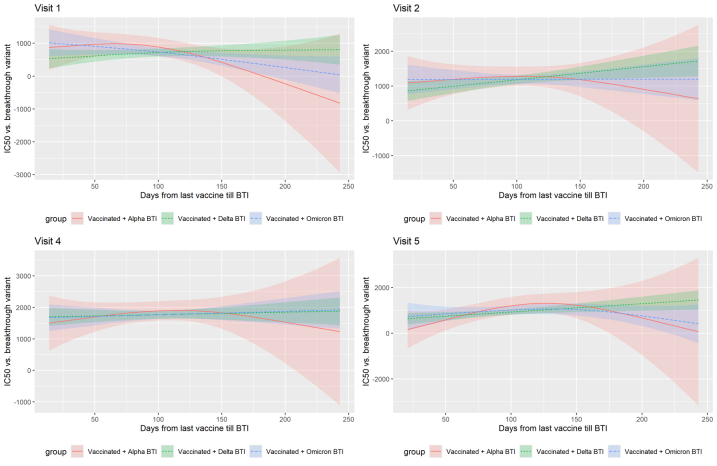
**Non-linear association of time intervals between vaccination and BTI on the nAb response (IC50) towards the infecting variant**. Generalized additive model smooths based on thin plate regression splines, with automatic selection of the effective degrees of freedom, for alpha (red, n = 24), delta (green, n = 92) and omicron (blue, n = 55) BTI. Shown are separate models for Visit 1, Visit 2, Visit 4 and Visit 5. Shaded regions indicate approximate 95%-prediction intervals (Prediction ± 2×SE).

Additionally, the effects of the time intervals between vaccination and BTI on the nAb response towards the infecting variant were investigated. Spearman correlation revealed significant but poor correlation only for V2 and V5 (p = 0.030, rho = 0.165 and p = 0.002, rho = 0.238, respectively). Subsequent nonlinear regression models exhibited non-significant or insufficient fits for all visits. Overall, only a maximum of 9.39% of the individual IC50 deviance could be explained by the best model (for visit 5). Breakthrough variant stratified analysis of did not reveal any significant smooths (Supplementary Figure S6).

### Magnitude-breadth analysis of alpha-/delta-/omicron-infected groups

**Fig. 2:**
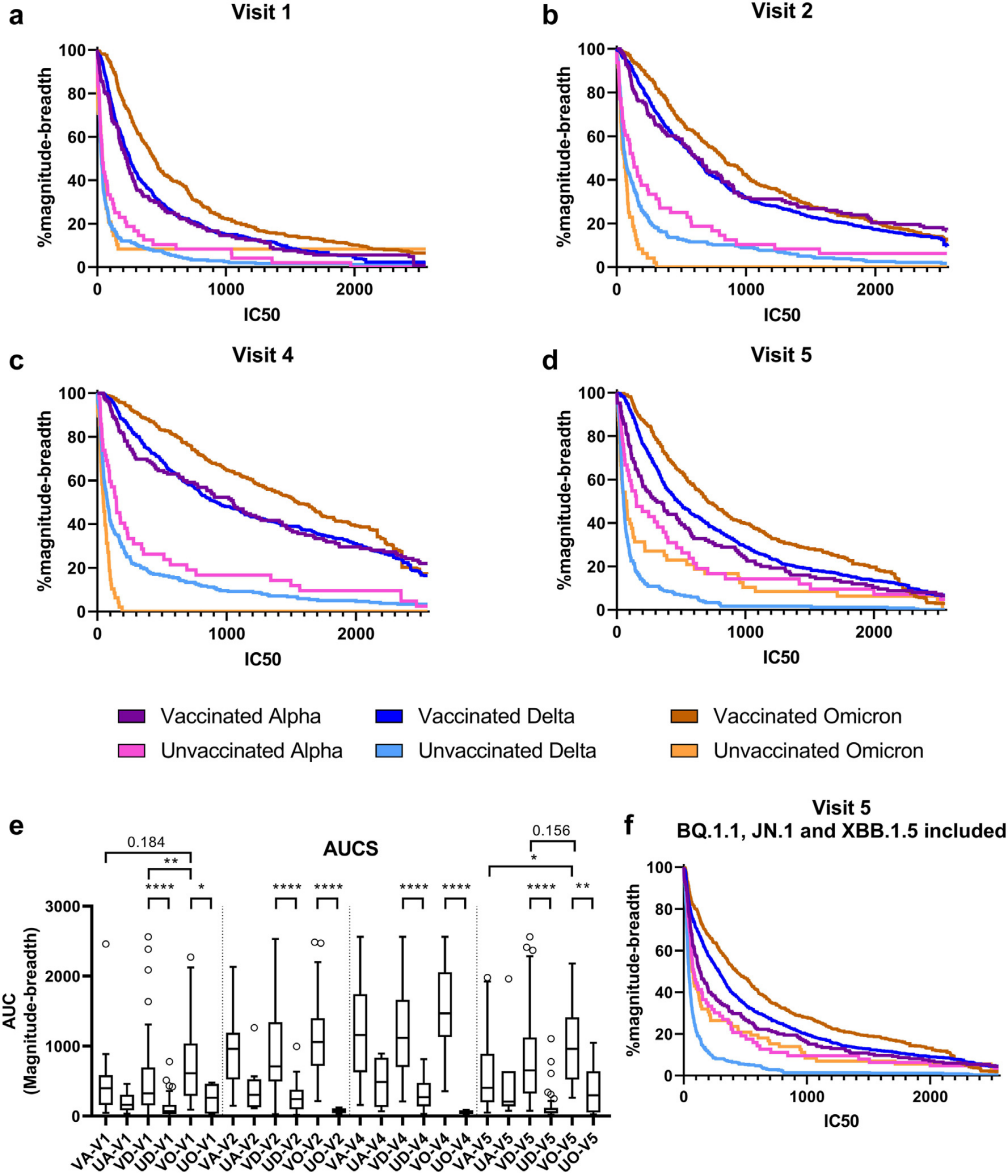
**Magnitude-breadth analysis for all study groups at the different visits**. Magnitude-breadth curves of the vaccinated & Alpha infected (VA, n = 24), unvaccinated Alpha infected (UA, n = 8), vaccinated & Delta infected (VD, n = 92), unvaccinated Delta infected (UD, n = 40), vaccinated & Omicron infected (VO, n = 55) and unvaccinated Omicron infected (UO, n = 8) groups at visit 1 (V1), visit 2 (V2), visit 4 (V4) and visit 5 (V5) (**a–d**). **e** Magnitude-breadth curves at V5 with the variant BQ.1.1 included in the analysis. **f** Calculated Areas under the curve (AUC) of the magnitude-breadth curves in **a-d** for each infection variant-vaccination status combination sorted by visits. Statistically significant differences are indicated by p values (∗p < 0.05, ∗∗p < 0.005, ∗∗∗p < 0.001, ∗∗∗∗p < 0.0001) [Kruskal–Wallis test and post-hoc tests adjusted by Dunn's correction for multiple tests].

To explore and visualize differences in the magnitude and breadth of neutralizing antibodies responses, infection variant and vaccination-status stratified (U = unvaccinated, V = vaccinated; A = Alpha infection, D = Delta infection, O = Omicron infection) magnitude-breadth curves were calculated for the study visits V1, V2, V4 and V5 (Fig. 2a–d). The corresponding AUCs were determined based on the nAb titres against WT/D614G, Alpha, Delta, Omicron BA.1, BA.2, and BA.5, respectively (Fig. 2e).

In line with the analysis of single variant neutralization, vaccinated groups revealed consistently higher magnitude-breadth than unvaccinated groups between visits V1 and V4 (Fig. 2a–c, e). While the unvaccinated groups showed very low magnitude-breadth profiles at V1, higher and comparable magnitude-breadth was observed in all vaccinated groups (Fig. 2a–e). Whereas the magnitude breadth profiles of all unvaccinated groups remained at overall low levels (V2–V4) ([Friedman test] V1–V4 p (UA) = 0,6529, p (UD) < 0.0001, p (UO) = 0,1993), a significant increase was found for all vaccinated groups, respectively ([Friedman test] V1–V4 p (VA) < 0.0001, p (VD) < 0.0001, p (VO) < 0.0001) (Fig. 2b, c, e). At visit 1, significantly increased magnitude-breadth was found for vaccinated omicron infected compared to vaccinated individuals with delta BTI ([Kruskal–Wallis with Dunn's] p < 0.01) yet not for alpha BTI ([Kruskal–Wallis with Dunn's] p = 0.184). Else, until V4 no significant variant-specific differences in magnitude-breadth levels were observed neither in the vaccinated nor the unvaccinated groups (Fig. 2e).

In contrast, the long-term visit V5 revealed significant differences ([Kruskal–Wallis] V5 p < 0.0001) between variant stratified groups. Alpha BTIs lost neutralization magnitude and breadth ([Friedman-Dunn's] p (VA V4 vs. VA V5)<0.0001) and yielded significantly lower AUCs compared to Omicron BTIs ([Kruskal–Wallis Dunn's] p (VA V5 vs. VO V5)<0.05), dropping down to the unvaccinated groups level (Fig. 2d and e). Though magnitude-breadth profiles for Delta and Omicron BTIs also significantly decreased compared to V4 ([Friedman Dunn's] p (VD V4 vs. V5)<0.0001; p (VO V4 vs. V5) <0.0001), the magnitude-breadth AUC remained significantly higher compared to unvaccinated groups. At V5, Omicron BTIs showed though not significant compared to Delta BTI, the highest median neutralization magnitude-breadth AUC of all groups ([Kruskal–Wallis Dunn's] p (VO vs. VD = 0.156), p (VA vs. VO < 0.05)), which was also seen when including neutralization data for Omicron BQ.1.1 (Fig. 2f).

**Supplementary Figure S7:**
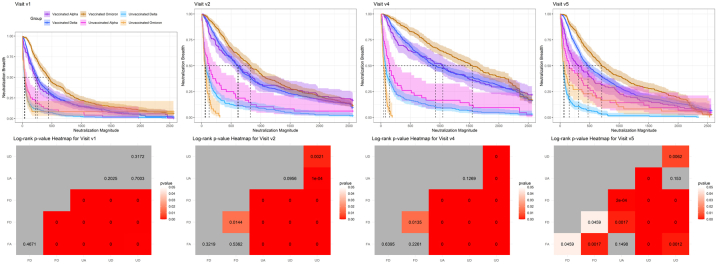
**Magnitude breadth curves with confidence 95% confidence intervals and log-rank tests as alternative comparison method**. Shown are the four panels (one for each visit) with magnitude breadth curves colour coded for the different groups (Purple = Alpha BTI n = 24, Dark blue = Delta BTI n = 92, brown = Omicron BTI n = 55 and pink = Alpha infection n = 8, light blue = Delta infection n = 40, orange = Omicron infection n = 8). Below are the results of the log rank tests for comparison of all groups (FA = Alpha BTI, FD = Delta BTI, FO = Omicron BTI and UA = Alpha infection, UD = Delta infection, UO = Omicron infection), plotted as a heatmap (grey = not significant, white to red = significant, 0 denotes a p value < 0.0001) for each visit. [log-rank-test].

To provide an additional layer of comparison, log-rank tests were performed to compare the overall magnitude breadth curves (Supplementary Figure S7), confirming our AUC-based comparison of curves. At visit 1–4, significantly higher magnitude breadth was found for all vaccinated groups compared to the unvaccinated groups ([log-rank] all p < 0.0001). At visit 1, Omicron BTI exhibited even higher initial magnitude breadth than Delta or Alpha BTI ([log-rank] both p < 0.0001). At visit 2 and 4, significantly reduced magnitude breadth was found for Omicron infection without vaccination compared to Alpha or Delta infection ([log-rank] all p < 0.0021). As opposed to comparing AUCs, Omicron BTI elicited a significantly higher magnitude breadth compared to Delta BTI ([log-rank] visit 2 p = 0.0144, visit 4 p = 0.0135). At visit 5, Omicron BTI still exhibited a significantly higher magnitude breadth than Delta BTI ([log-rank] p = 0.0459) and Alpha BTI ([log-rank] p = 0.0017), while Delta BTI exhibited a significantly increased magnitude breadth compared to Alpha BTI (p = 0.0459). Surprisingly, magnitude breadth of Omicron infected without a vaccination increased at visit 5 to similar levels as Alpha infection, leaving delta infection in the last place ([log-rank] p Alpha vs. Delta <0.0001, p Omicron vs. Delta = 0.0062).

**Supplementary Figure S8:**
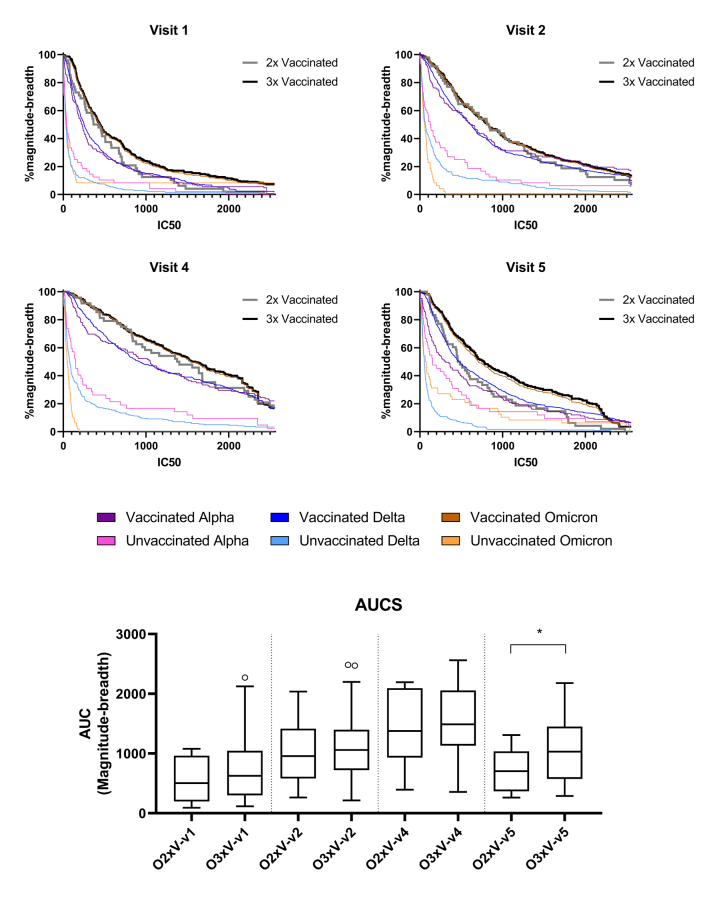
**Comparison of magnitude-breadth analysis between double- and triple-vaccinated Omicron groups**. To the magnitude breadth curves of Figure 2 the curves for double- (O2xV n = 48) and triple-(O3xV, n = 7) vaccinated & Omicron infected subgroups are added at visit 1 (V1), visit 2 (V2), visit 4 (V4) and visit 5 (V5). Area under the curve (AUC) for the double- and triple-vaccinated & Omicron infected groups are calculated and compared at V1, V2, V4 and V5. Statistically significant differences are indicated by p values (∗ p < 0.05, ∗∗ p < 0.005, ∗∗∗ p <0.001)[Mann-Whitney U].

Noteworthy, a subgroup analysis of the two-vs. three-time-vaccinated individuals with Omicron BTI (Supplementary Figure S8) at V5 revealed that the magnitude breadth level of triple-vaccinated individuals remained significantly higher in comparison to 2x vaccinated individuals ([Mann–Whitney] p (O3xV vs. O2xV) = 0.0384) on the level of the previously analysed vaccinated Omicron BTI group (Supplementary Figure S8). The 2x vaccinated individuals with Omicron BTI dropped to levels similar to those of 2x vaccinated individuals with Delta BTI. Like the previous analysis, no significant differences regarding magnitude and breath were found in 2x or 3x vaccinated between V1 and V4 (Supplementary Figure S8).

### Multivariate analysis

Multivariate analysis was conducted using separate PERMANOVA models for individuals with vaccination as well as individuals without vaccination, this enabled inclusion of vaccine-specific variables such as days between vaccination and infection, number of vaccinations, and if the individual received any vector vaccine. Those were included on top of sex/gender, age, smoking status, neutralized variant, and infection variant as used for modelling individuals without vaccination.

For vaccinated individuals, we found significant impact of age (p = 0.001), number of vaccinations (p = 0.009), time between vaccination and infection (p = 0.001), virus to be neutralized (p = 0.001), and the infection variant (p = 0.001) on neutralization. Post-hoc pairwise PERMANOVA testing adjusted for all aforementioned variables utilizing tobit models identified significant differences between Omicron and Delta BTI (p = 0.006) as well as Omicron and Alpha BTI (p = 0.015) but no differences between Delta and Alpha BTI (p = 0.158).

Similarly, significant impact of the infection variant (p = 0.001) and virus to be neutralized (p = 0.001) was found for individuals without vaccination before infection. In contrast to vaccinated individuals, age did not have an impact in unvaccinated individuals (p = 0.978) but a significant impact was found for smoking (p = 0.005). Adjusted post-hoc pairwise PERMANOVA testing revealed significant differences between all cross-compared groups: Delta, Omicron and Alpha infection (all p = 0.003).

To further explore the differences between individuals with or without vaccination, we utilized a tobit-model (modelling neutralization against gender, age, nicotine consumption, infection variant, vaccination status, neutralized virus and visit, with interaction effects of infection variant and vaccination status) to adjust for age, gender and nicotine consumption. Here, the model identified significant positive effects for age (p = 0.0031) and negative effects for nicotine consumption (p (yes) = 0.0028), whereas gender didn't show significant impact. Of note, the model already identified a highly significant positive impact of vaccination (p < 0.001). After adjustment, PERMANOVA revealed highly significant influence (p < 0.001) of vaccine status, neutralized virus, and infection variant, whereas pairwise post-hoc PERMANOVA revealed highly significant differences between vaccinated and unvaccinated individuals (p < 0.001) with an R^2^ of 0.149.

### Antigenic mapping and antibody landscapes

**Fig. 3:**
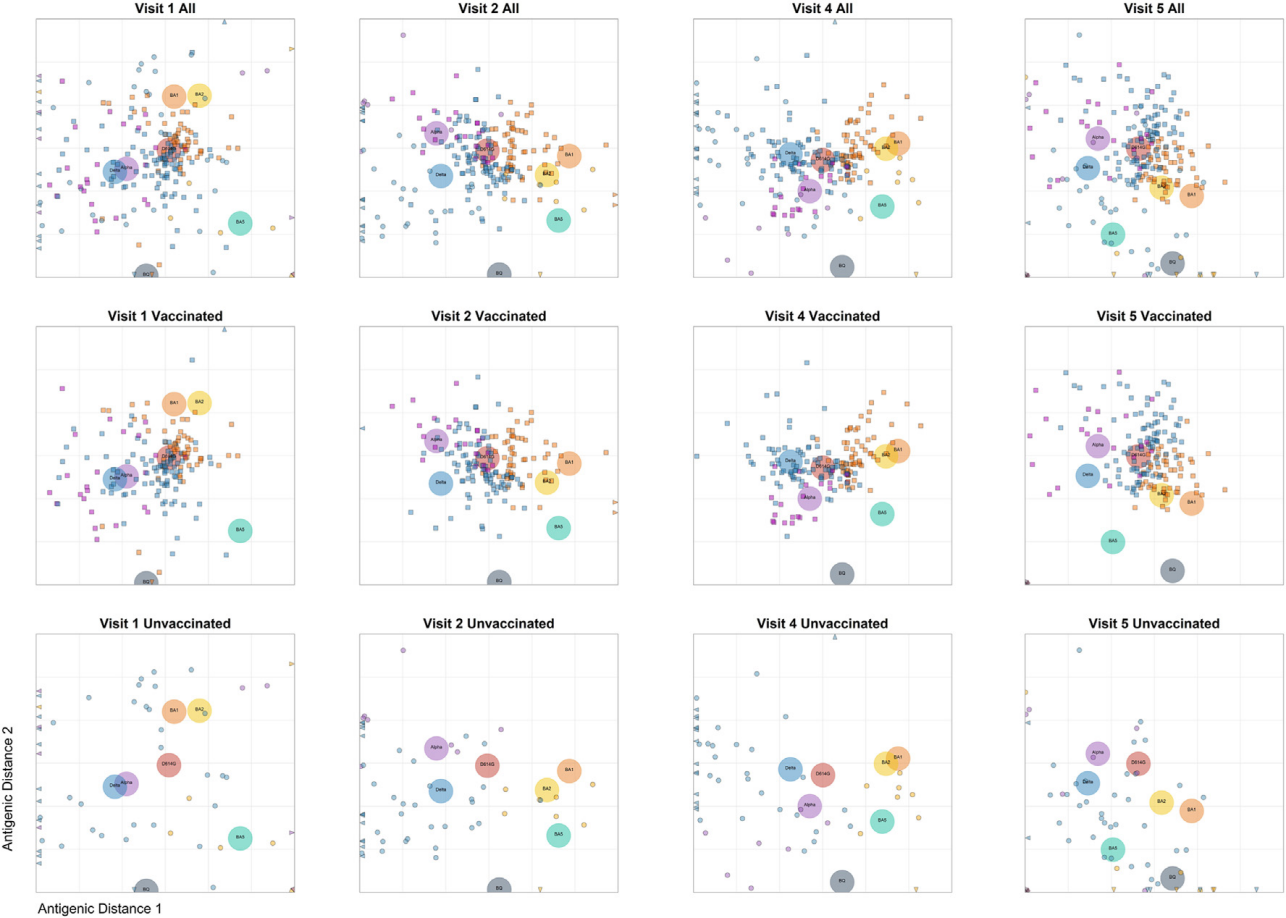
**Antigenic maps for SARS-CoV-2 variants**. Antigenic maps for four different study time points (V1, V2, V4 and V5) based on sera of vaccinated (n = 171) and unvaccinated (n = 56) participants with Alpha, Delta or Omicron (n = 32, 132, 63, respectively) infections. The SARS-CoV-2 variants are shown as big, coloured circles on the map Wuhan/D614G (red), Alpha (purple), Delta (blue), Omicron BA.1 (orange), BA.2 (yellow), BA.5 (green) and BQ.1.1 (grey) and respective individual sera are indicated as small squares (vaccinated) or small circles (unvaccinated) in purple, blue and orange for Alpha, Delta or Omicron infections, respectively. The y- and x-axes of the map both represent antigenic distance. Each grid square (1 antigenic unit) represents a 2-fold change in neutralization titre. Row one are maps including all sera, while rows two and three show only the sera of vaccinated or unvaccinated individuals, as indicated.

Subsequently, we used our neutralization data to construct antigenic maps, visualizing the antigenic relationship of the tested viruses and antibody responses elicited in the different groups, for all study time points (Fig. 3). The mapped distances between VOCs and the relative placement of the individual antisera, respectively, correspond to the antigenic relationship and indicate the profile of the antibody response for the respective groups at the indicated time points (V1-5).

At early convalescence (V1–V2), the sera of the vaccinated groups cluster mainly around the vaccine-matched WT/D614G strain, regardless of the infecting VOC. This suggests that vaccine-primed nAb specificities are extended in the first place. Beginning at V2 and towards the peak of the neutralizing antibody responses at V4, the sera of the vaccinated groups shift with time towards the respective infecting variant (e.g. sera from Alpha BTI shifting towards the Alpha variant). Interestingly, in the long-term (V5) the sera from all BTI groups revoked and shifted back towards the vaccine-matched WT/D614G strain (Fig. 3).

**Supplementary Figure S9:**
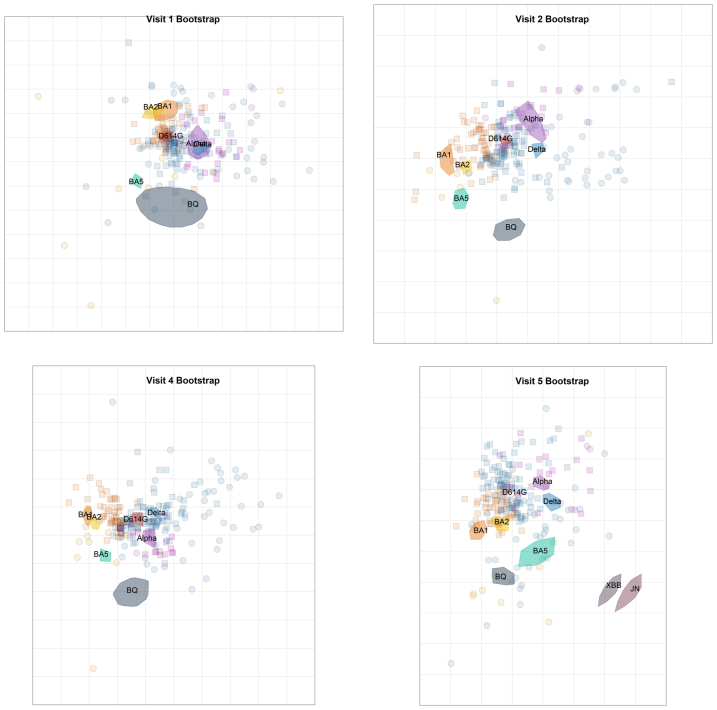
**Assessing the effect of the uncertainty in variant reactivity for the antigenic maps using bootstrapping**. Antigenic maps for four different study time points (V1, V2, V4 and V5, as indicated) based on sera of vaccinated and unvaccinated participants with Alpha, Delta or Omicron infections. The SARS-CoV-2 variants are shown as big coloured circles on the map WT/D614G (red), Alpha (purple), Delta (blue), Omicron BA.1 (orange), BA.2 (yellow), BA.5 (green) and BQ.1.1. (grey) Individual sera are not shown. The x- and y-axes of the map both represent antigenic distance. Each grid square (1 antigenic unit) represents a 2-fold change in neutralization titre. 1000 resampling bootstrap repeats were performed with 100 optimizations per repeat. at all visits. Each coloured region indicates the area in which 68% (one standard deviation) of the positional variation of variant.

In contrast, the sera from unvaccinated individuals were initially more widely distributed at V1, mainly due to initially very low neutralization titres, but at the later visits increasingly clustered around the infecting variant, most pronounced at peak neutralizing responses at V4. At V5, the localizations of the unvaccinated sera dispersed again, likely due to the lower overall neutralization titres (Figs. 3 and 1). Bootstrapping and calculation of confidence intervals confirmed the localization of virus variants on the map, while the highest insecurity was found for BQ.1.1 at V1, and the highest confidence levels were achieved at V4 (Supplementary Figure S9).

**Fig. 4:**
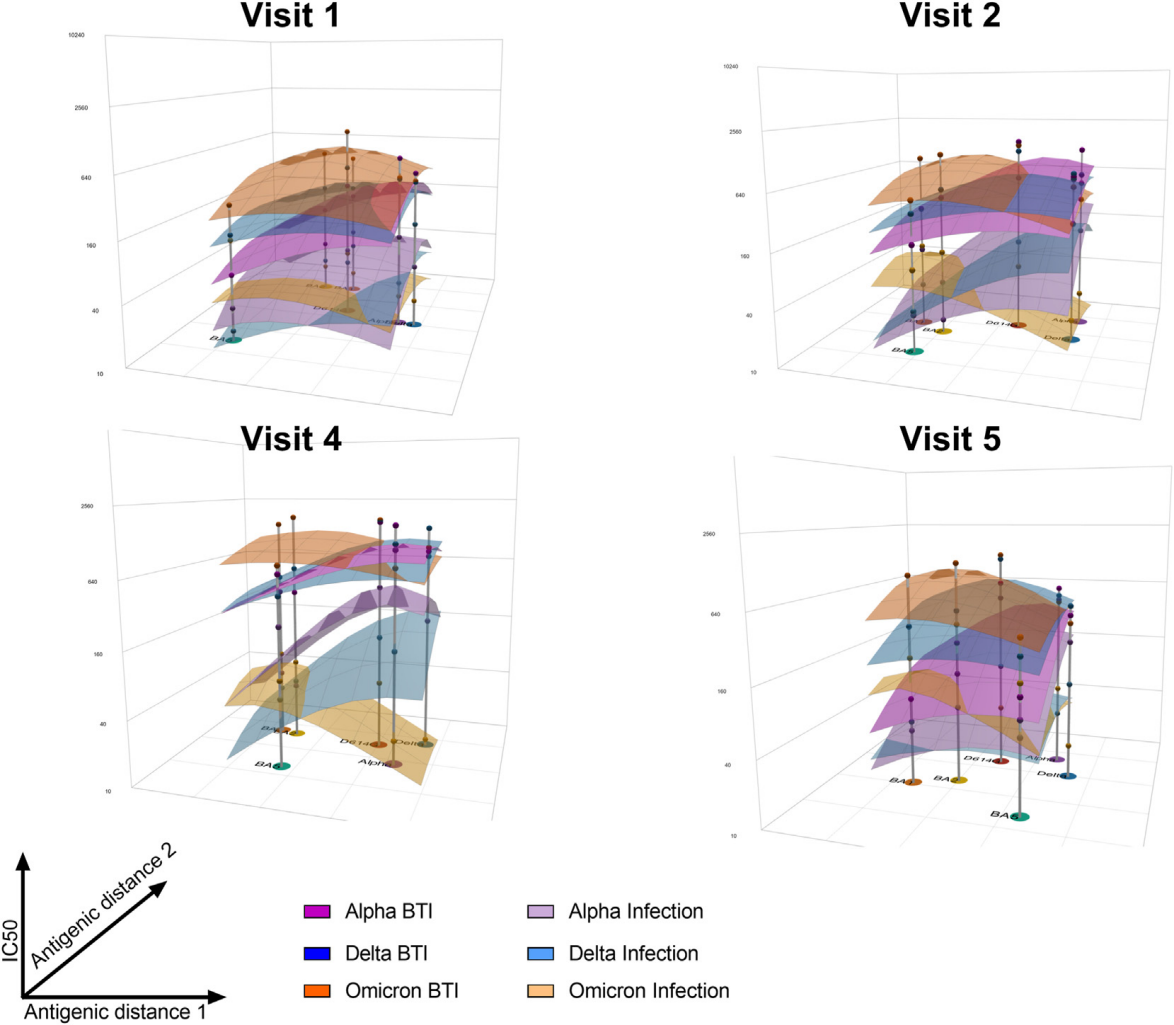
**Antibody landscapes for each study group**. Coloured surfaces show the fitted geometric mean titre (GMT) antibody landscapes for the different study groups vaccinated & Alpha infected (dark purple, n = 24), unvaccinated Alpha infected (light purple, n = 8), vaccinated & Delta infected (dark blue, n = 92), unvaccinated Delta infected (light blue, n = 40), vaccinated & Omicron infected (dark yellow, n = 55) and unvaccinated Omicron infected (light yellow, n = 8). While the base x-y plane corresponds to the antigenic map shown in Fig. 3, grey impulses show the height of the GMT for specific SARS-CoV-2 variants. The vertical z-axis in each plot corresponds to the GMT on the log2 scale, each two-fold increment is marked, starting from a titre of 20 at the map surface. The antibody landscapes for visit 1 can be seen in the upper left panel, visit 2 in the upper right panel, visit 4 in the lower left panel and visit 5 in the lower right panel.

**Supplementary Figure S10:**
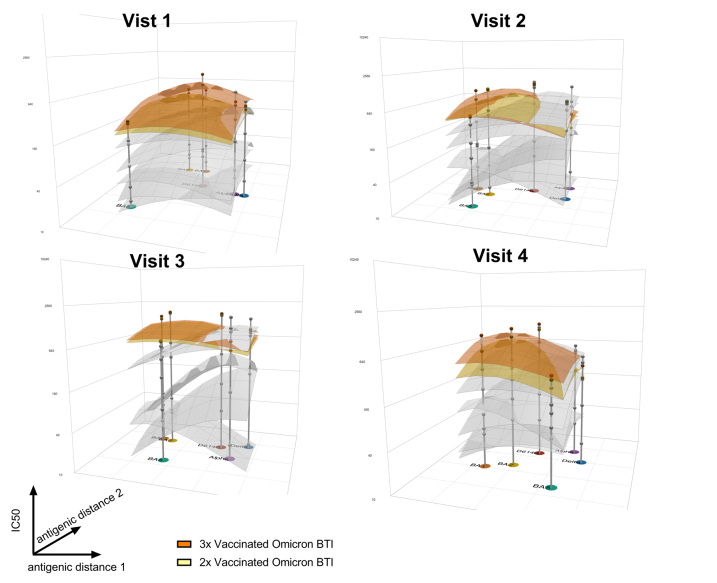
**Antibody landscapes for Omicron double-vaccinated versus triple-vaccinated cases**. Coloured surfaces show the fitted geometric mean titre (GMT) antibody landscapes for the different study groups vaccinated & Alpha infected (grey), unvaccinated Alpha infected (grey), vaccinated & Delta infected (grey), unvaccinated Delta infected (grey), triple vaccinated & Omicron infected (dark yellow, n = 48), double vaccinated & Omicron infected (red, n = 7) and unvaccinated Omicron infected (grey). While the base x-y plane corresponds to the antigenic map shown in Figure 3, grey impulses show the height of the GMT for specific SARS-CoV-2 variants. The vertical z-axis in each plot corresponds to the GMT on the log2 scale, each two-fold increment is marked, starting from a titre of 20at the map surface. The antibody landscapes for visit 1 can be seen in the upper left panel, visit 2 in the upper right panel, visit 4 in the lower left panel and visit 5 in the lower right panel.

Finally, antibody landscaping complemented antigenic mapping by the addition of quantitative neutralization information. Confirming our magnitude-breadth analysis, we found an increased and broadened response in vaccinated vs. unvaccinated individuals at V1–V4 (Fig. 4). Similar shapes and magnitudes were calculated for the vaccinated groups at V1 and V2. In contrast, obvious differences amongst the vaccinated groups were revealed at peak response (V4). This is particularly evident comparing the tilt of the Omicron BTI group displaying a peak at the map's BA.1/2 cluster with the Delta infected individuals peaking at the map's Alpha/Delta cluster (Fig. 4). This was true for both, the two times and three times vaccinated after Omicron BTI (Supplementary Figure S10). At V5 tilting reverted back towards a peak at the WT/D614G strain, leaving similar landscapes for both, Delta and Omicron BTI. Additionally, sustained neutralization magnitude and breadth were found after Omicron or Delta BTI, while Alpha BTI reverted back towards the levels of unvaccinated groups (Fig. 4).

### Machine learning models to predict infecting VOC from neutralization profiles

**Fig. 5:**
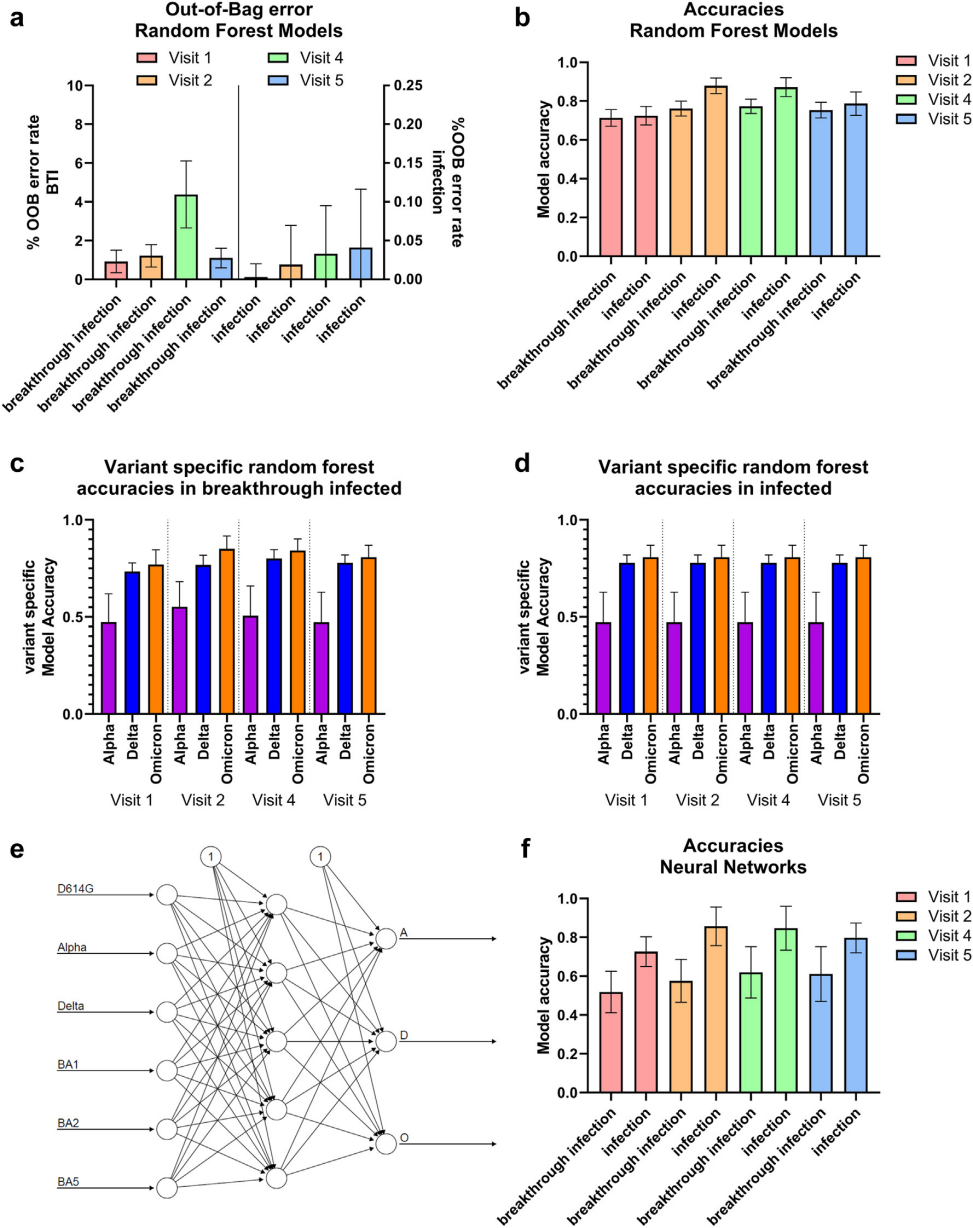
**Machine learning model accuracy and error rates**. **a** Out of bag error rate of random forest models for the vaccinated, breakthrough infected and unvaccinated, infected groups at the visits 1,2,4 and 5. **b** Random forest model accuracies for the vaccinated and breakthrough infected or unvaccinated and infected groups at the visits 1,2,4 and 5 on an independent test dataset. **c** Variant-specific accuracy in vaccinated and breakthrough infected individuals for visit 1,2,4 and 5 **d** Variant-specific accuracy in unvaccinated and infected individuals for visit 1,2,4 and 5. **e** Exemplary neuron configuration of the neural networks used. With 6 input neurons, one hidden layer of 5 neurons and three output neurons giving the probability for A (Alpha), D (Delta) and O (Omicron) infection. **f** Neural network model accuracies for the vaccinated and breakthrough infected or unvaccinated and infected groups at the visits 1,2,4 and 5 on independent test datasets (n (vaccinated) = 171, thereof 24 Alpha, 92 Delta and 55 Omicron; n (unvaccinated) = 56, thereof 8 Alpha, 40 Delta, 8 Omicron, each n simulated to 333 as described in the methods, model replicates (Random Forest) = 10,000, model replicates (neural networks) = 100).

To confirm expansion of the nAb profile towards the break-through infecting variant, random-forest models were trained to predict the infecting VOC (Alpha, Delta, Omicron) from the neutralization profile in break-through infected or infected individuals. Reported are mean accuracies and errors of 100 models trained and tested on independently generated datasets. Out-of-bag error was at 4.38% for the vaccinated at V4 and below 1.1% for all other conditions, showing solid training for all models (Fig. 5a).

In unvaccinated individuals, model accuracy increased from 72.5% (visit 1) to 87.8 and 87.1% for V2 and V4, respectively, staying at a high level of 78.7% for the long-term visit (V5). Similarly, model accuracy in vaccinated individuals started with 71.3% (V1), increased to 76.1% (V2), peaked at V4 (77.3%) and slightly decreased towards the long-term V5 (75.3%). Model accuracies for the break-through infected were slightly reduced compared to the unvaccinated group. However, overall prediction of the infecting variant was still reliable in all BTI models, especially at V4. This provides additional proof, of a strong shift in overall immune profiles following BTI - despite prior vaccination (Fig. 5b).

Consistently lowest variant-specific accuracy (25–70%) was found for Alpha BTI throughout all visits, slightly increasing towards visit 2 and 4. For Delta and Omicron, increased accuracies were found for V2 and V4, staying high at the long-term V5, similar to the general model accuracy. Highest accuracies were achieved for omicron BTI. A similar pattern was found for the unvaccinated and infected (Fig. 5c and d).

Random forest results were independently verified by training neural networks as exemplarily shown in Fig. 5e, achieving accuracies of 51.8, 57.6, 61.9 and 61.1% for the breakthrough infected individuals and 72.6, 85.7, 84.7 and 79.7% in the infected individuals for the respective visits (Fig. 5f).

## Discussion

Our study demonstrated that hybrid immunity, i.e. the immune response acquired by vaccination and subsequent BTI is superior to infection-induced immunity alone in terms of magnitude, breadth, and durability of the neutralizing immune response. Yet, an antigenically closely related immune response of limited magnitude and breadth, may still be superior against a newly emerging VOC, compared to an antigenically distant immune response of high magnitude and breadth. The highest magnitude and breadth were observed in BTIs with the VOCs Delta and Omicron, which are antigenically most distant to the vaccine-encoded wildtype-based SARS-CoV-2 spike. Nonetheless, even those groups lost their long-term response against novel and far distant variants like XBB1.5. and JN.1, whereas Omicron infection without prior vaccination revealed a significantly higher response against those far distant Omicron descendants as compared to delta infection. Noteworthy, a third vaccination, although based on the original SARS-CoV-2 wildtype spike, significantly improved the long-term magnitude-breadth profile, while we didn't find any impact of a vector vaccine history. Antigenic mapping indicated specific neutralizing antibody profiles elicited by BTI or non-BTI, which evolved differently depending on the infecting SARS-CoV-2 VOC. Noteworthy this analysis provided evidence of initial immune imprinting by vaccination prior to BTI with any of the VOC. Finally, antigenic landscaping highlighted the impact of a third vaccination and helped to quantitatively refine our finding of a long-term bias towards immunity against the vaccine antigen.

Whereas the majority of previous studies on BTI are either based on smaller numbers of participants or lacking long-term follow-up data,^33^, ^34^, ^35^ our prospective and longitudinal multicentre study design comprised 229 participants, who were observed for a period of 4–6 months. A Kaplan–Meier function was introduced to quantify and combine neutralization against multiple SARS-CoV-2 VOCs (magnitude-breadth) at different time points. Our data confirm and expand previous findings showing that VOC BTIs enlarge the functional neutralization footprint of wild-type based COVID-19 vaccines.^36^^,^^37^

Previous studies have found differences in individuals vaccinated with mRNA-based vaccines compared to individuals receiving a combination of Adenoviral and mRNA-based vaccines, levelled out by a third vaccination.^38^ Our data suggest that those differences get largely levelled out by breakthrough infection, though increased titres against D614G and Alpha hint towards a slightly increased durability. However, regarding the heterogenicity and size of the vector-vaccinated group within this study-collective, those results should not be over-interpreted. A different cohort, designed around this research topic would be needed to provide more reliable insights. On top, our longitudinal design combined with modelling identified follow-up time after infection and breakthrough infection as well as study centre effects as potential confounding factors, which ought to be considered when conducting those kinds of studies. Of note, despite our efforts to account for those confounding factors in the vaccinated and unvaccinated individuals, we can't fully exclude additional infection-virus-group-specific effects, as group sizes became too small to adjust.

Previous data have shown,^36^ that short- or long-time intervals between vaccination and subsequent BTI impede or enlarge neutralizing antibody levels, respectively. Our data added evidence, that enlarged intervals barely influenced short-term nAb responses against the infecting VOC but had some positive impact on the long-term outcome of the neutralization capacity.

Furthermore, a third vaccination was demonstrated to have long-term benefit neutralization magnitude and breadth,^39^, ^40^, ^41^ even for more recent VOC such as BQ.1.1. Noteworthy, also the antigenic distance between the spike antigen presented with the BTI and the spike variant (WT/D614G) delivered by the vaccine significantly impacts the magnitude-breadth profile of hybrid immunity.^33^, ^34^, ^35^^,^^42^ In particular, our longitudinal analysis demonstrates that the long-term neutralization magnitude-breadth persisted at higher levels in Delta or Omicron BTI if compared to Alpha BTIs, which are antigenically most related to the COVID-19 vaccine. This suggests that a durable nAb response with a high magnitude-breadth is preferentially induced by exposure to antigenically more distant Delta or Omicron variants. Nonetheless, even those groups lost their long-term response against novel and far distant variants like XBB1.5. and JN.1, whereas Omicron infection without prior vaccination showed similar response against those far distant Omicron descendants compared to vaccination and Omicron BTI.

Using antigenic cartography, initially developed to evaluate the quality of neutralizing antibody responses against multiple influenza variants,^43^ the analysis of sera from unvaccinated infected individuals confirmed distinct antigenic clusters, formed dependent on the antigenic relationship of the analysed SARS-CoV-2 variants.^28^^,^^44^^,^^45^ Additionally, we found different antibody profiles depending on vaccination history and the infecting VOC. Noteworthy, the longitudinal evolution of antigenic maps in BTIs suggests an initial boost of the vaccine-primed wildtype-directed nAb response followed by a broadening of nAb responses towards the infecting variant with time. At the long-term visit, a subsequent contraction of the nAbs towards wild-type-specific nAbs dominating could be observed.

This effect might be explained by either de-novo stimulation of naïve B-cells or, alternatively, by affinity maturation and/or expansion of already primed memory B-cells. Recent evidence based on antibody depletion studies and longitudinal BCR sequencing convincingly suggests boosting and expansion of imprinted B-cell specificities as the most probable explanation for the observed broadening of neutralization capacity.^46^^,^^47^ This pattern hints towards a phenomenon referred to as “immune imprinting”, which has previously been found for other viruses such as influenza.^12^ Our data would also be compatible with a de novo priming of VOC-specific neutralizing B cell responses, peaking at V4, but vanishing faster as WT specific neutralization due to lack of expansion and affinity maturation. As evidenced recently, more sustained responses to the new and antigenically distant infecting variant could be achieved by repeated contact eventually overcoming immune imprinting.^48^ Both interpretations would be in agreement with our quantitative analysis highlighting that neutralization magnitude-breadth improved with antigenic distance of the VOC causing the BTI to the vaccine antigen. Further implications of those phenomenons are effectively illustrated with our data of an increased immune response towards novel Omicron descendants such as BQ.1.1, XBB.1.5. and JN.1^49^ in individuals with Omicron infection but no vaccination history. Emphasizing, that a lower overall breadth, but imprinted with an antigenically closer antigen may still be beneficial over a high overall breadth but imprinted with a distant antigen.

Finally, antibody landscaping^27^ affirmed and expanded our magnitude-breadth analysis by resolving the contribution of VOC specific neutralization. This was especially relevant comparing Omicron and Delta BTI, which yielded different slopes and directions in their fitted landscapes, especially when the three times vaccinated individuals were excluded. Subsequent reversion towards similar peaks and slopes after Delta or Omicron BTI regarding long-term nAb response provide further confirmation for the observed long-term bias towards WT SARS-CoV-2 neutralization. Random forest models and neural networks reliably predicted the infecting and, noteworthy, also the breakthrough infecting VOC from the neutralization profiles, including at the long term V5. This independently confirmed a sustained shift of the neutralization profile towards the breakthrough infecting VOC, despite prior vaccination and immune imprinting. Of note, multiple potential risks can be associated with machine learning-based data analysis, possibly resulting in biased results^50^(Preprint). We tried to minimize bias derived from sample imbalance by resampling and counteract potential bias from overfitting, by introducing noise into resampled values. Looking at the different variant-specific accuracies, a consistently lower Alpha accuracy implied, that those measures might positively influence but still not fully equalize the bias derived from small and imbalanced groups. This expresses the need for larger cohorts or even combined databases of multiple cohorts to yield more refined models. To minimize the impact of this potential bias and still allow careful interpretation, we reported mean accuracies of 100 independent models on 100 independently drawn datasets. Another relevant consideration is the neural network's lower predictive accuracy, despite the neural networks' theoretical advantages in modelling complex, non-linear relationships. This highlights their potential underperformance in smaller datasets, once again underscoring the necessity of larger datasets and maybe necessary inclusion of additional variables for more refined models. Nevertheless, we think our work provides a solid proof of concept for the usability of such models in answering arising questions of the field.

Our study also has some limitations. Due to the lack of pre-infection samples and analytical reference points, we could not exactly determine the impact of preexisting immunity. Furthermore, the comparably low number of individuals in the unvaccinated Alpha and Omicron groups could impact comparisons between non-BTIs and BTIs, respectively. Nevertheless, the results shown align with the results obtained for comparing Delta infection with and without prior vaccination. Another possible source of bias might derive from cohort differences, in specific, significantly increased prevalence regarding preexisting disease in individuals with vaccination compared to unvaccinated individuals. However, no significant differences were found regarding immune-related disease.^21^ Furthermore, despite random and multicentre recruitment of acutely infected individuals with and without prior vaccination reported by health authorities, we cannot fully exclude potential sampling bias e.g. due to individuals avoiding official SARS-CoV-2 testing to evade strict quarantine regulations or other confounding effects. Finally, unknown demographics of the infected population with or without vaccination at the time of sampling and thus unknown representativeness should be considered when interpreting the study results. Moreover, there is uncertainty because of low statistical power for certain subgroups formed ex post, such as unvaccinated omicron BTI, so that effects may be undetectable in these cases.

In conclusion, our prospective and longitudinal multicentre cohort study revealed that neutralization capacity, breadth, and durability did benefit from the number of vaccinations and antigenic distance between the infecting VOC and the WT spike delivered via the vaccine. Antigenic mapping, antibody landscaping, and machine learning models affirmed distinct immune profiles, shaped based on the infecting VOC and time after BTI. Our longitudinal study design proved an immune imprinted initial boost of vaccine-specific neutralization and a delayed shift towards the infecting VOC at peak response, while the long-term neutralization was contracted towards the vaccine antigen.

## Contributors

Conceptualization: R.W., K.Ü., P.S.; Methodology: S.E., R.W., Participant recruitment and study visits: S.D.J., C.A., A.F., M.W., S.B., C.J., C.C., A.Wil., M.V., M.We., B.M.J.L., V.S., B.L., P.S., CoVaKo study group; Laboratory investigations: S.E., A.S., D.P.; Statistical analysis: S.E., B.A., R.W. Visualization: S.E., C.A., P.S.; Resources: M.P., H.M., U.P., J.L., M.H., R.W., K.Ü.; Writing (original draft): S.E., C.A., R.W.; Writing (review and editing): all authors; Accessed and verified the underlying data: S.E., C.A., R.W.; Supervision: M.P., H.M., U.P., J.L., M.H., R.W., K.Ü., P.S.; Project administration: K.Ü., P.S.; Funding acquisition: H.M., U.P., J.L., M.H., R.W., K.Ü.; All authors have read and agreed to the published version of the manuscript.

## Data sharing statement

A github repository containing the dataset and all the code is available on github via https://github.com/rwagnerlab/Einhauseretal2024_imprinting_neutralizing_antibodies/.

## Declaration of interests

M.P. receives honoraria for scientific talks from Abbvie, BioNTech, Chugai-Roche, GSK, Esanum, Janssen, Novartis, Moderna, MSD, Pfizer, Sanofi, and SOBI, for consultant tasks from Abbvie, BioNTech, GSK, Janssen, Novartis, and Pfizer, travel scholarships from Chugai-Roche, GSK, Novartis, and Pfizer and support for investigator-initiated research from Baxter, Chugai-Roche, Galapagos, GSK, MSD, Moderna, Novartis, Pfizer and SOBI. U.P. received personal fees from Abbott, Abbvie, Arbutus, Gilead, GSK, J&J, MSD, Roche, Sanofi, Sobi, and Vaccitech. U.P. is co-founder, shareholder and board member of SCG Cell Therapy Inc. The CoVaKo consortium was funded by the Bavarian State Ministry of Science and the Arts. The funder had no influence on the study design, data analysis or data interpretation.

## Figures and Tables

**Validation-cellline:**
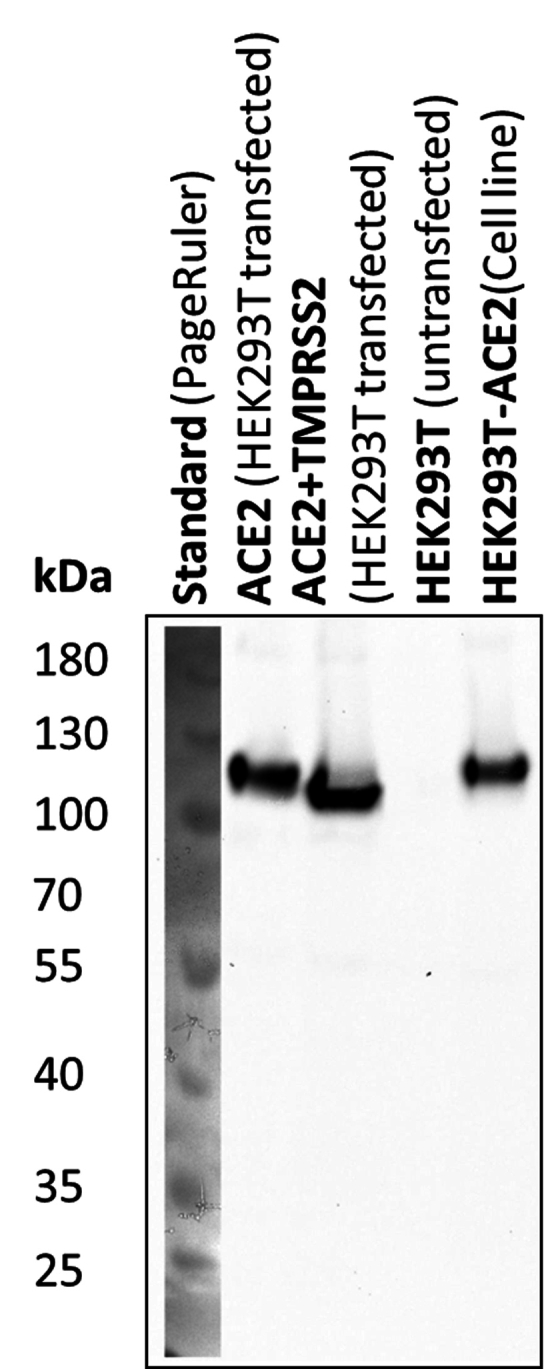


## Appendix A Supplementary data

Supplementary Tables

CoVaKostudygroup
