# Supplementary material for: Longitudinal effects of SARS-CoV-2 breakthrough infection on imprinting of neutralizing antibody responses
Source: eBioMedicine. 2024 Nov 9;110:105438. doi: 10.1016/j.ebiom.2024.105438 (PMC11585733; doi:10.1016/j.ebiom.2024.105438)
Supplement: Supplementary Tables [file mmc1.docx]

**Supplemental Table 1: Vaccination schemes of study cohorts**
