# Supplementary material for: Longitudinal effects of SARS-CoV-2 breakthrough infection on imprinting of neutralizing antibody responses
Source: eBioMedicine. 2024 Nov 9;110:105438. doi: 10.1016/j.ebiom.2024.105438 (PMC11585733; doi:10.1016/j.ebiom.2024.105438)
Supplement: CoVaKostudygroup [file mmc2.docx]

**Members of the CoVaKo study group**

| **Title & First Name** | **Last Name** |
| --- | --- |
| Prof. Dr. Helmut | Messmann |
| Dr. Andre | Fuchs |
| Dr. Alanna | Ebigbo |
| Dr. Christoph | Römmele |
| Maximilian | Ullrich |
| Marie | Freitag |
| Prof. Dr. Claudia | Traidl-Hoffmann |
| Mehmet | Goekkaya |
| Aline | Metz |
| Corinna | Holetschek |
| Prof. Dr. Avidan | Neumann |
| Prof. Dr. Reinhard | Hoffmann |
| Elisabeth | Kling |
| Mihail | Pruteanu |
| PD Dr. Thomas | Wibmer |
| Dr. Susanne | Rost |
| Prof. Dr. Klaus | Überla |
| Dr. Philipp | Steininger |
| Dr. Monika | Wytopil |
| Stephanie | Beileke |
| Dr. Sandra | Müller-Schmucker |
| Dr. Klaus | Korn |
| Tamara | Hastreiter |
| Kirsten | Fraedrich |
| Debora | Obergfäll |
| Dr. Frank | Neumann |
| Dr. Claudia | Kuhn |
| Dr. Katja | Günther |
| Dr. Elke | Friedrich |
| Prof. Dr. Michael | Hoelscher |
| PD Dr. Andreas | Wieser |
| PD Dr. Christof | Geldmacher |
| Christian | Janke |
| Michael | Plank |
| Jessica | Guggenbühl |
| Christina | Reinkemeyer |
| Ivan | Noreña |
| Dr. Noemi | Castelletti |
| Dr. Raquel Rubio | Acero |
| M. I.M. | Ahmed |
| Paulina | Diepers |
| Tabea M. | Eser |
| Anna | Fuchs |
| Olga | Baranov |
| Bernadette | Bauer |
| Danni | Wang |
| Ivana | Paunovic |
| Prof. Dr. Ulrike | Protzer |
| Samuel D. | Jeske |
| Catharina | Christa |
| Kathrin | Tinnefeld |
| Martin | Vu |
| Annika | Willmann |
| Dr. Hedwig | Roggendorf |
| Dr. Nina | Körber |
| Dr. Tanja | Bauer |
| PD Dr. Sabine | Gleich |
| Prof. Dr. Ralf | Wagner |
| Dr. Claudia | Asam |
| Sebastian | Einhauser |
| Manuela | Weps |
| Antonia | Senninger |
| Dr. George | Carnell |
| Prof. Jonathan Luke | Heeney |
| Antonia | Ebner |
| Maria | José de Schultz |
| Cedric | Rajes |
| Aya | Al Wafai |
| David | Brenner |
| Laura | Sicheneder |
| Melanie | Berr |
| Anja | Schütz |
| Dr. Stilla | Bauernfeind |
| Dr. Andreas | Hiergeist |
| Prof. Dr.Dr. André | Gessner |
| Prof. Dr. Barbara | Schmidt |
| Dr. Hans-Helmut | Niller |
| Dr. Jürgen | Wenzel |
| Daniela | Biermeier |
| Dr. Benedikt | Lampl |
| Ulrich | Rothe |
| Dr. Ute | Gleißner |
| Dr. Susanne | Brückner |
| Michaela | Treml |
| Holger | Schedl |
| Dr. Beate | Biermaier |
| Markus | Achatz |
| Dr. Daniela | Hierhammer |
| Johanna | Englhardt |
| Werner | Scheidl |
| Dr. Sivaji | Jeyaraman |
| Dr. Barbara | Schutt |
| Prof. Dr. Johannes | Liese |
| Prof. Dr. Martina | Prelog |
| PD Dr. Giovanni | Almanzar |
| Valeria | Schwägerl |
| Dr. Julia | Bley |
| Tim | Vogt |
| Kimia | Kousha |
| Lars | Ziegler |
| Astrid | Stein |
| Franziska | Förg |
| Dr. med. Johann | Löw |
| Barbara | Finkenberg |
| Dennis | Pollak |
| Alexander | Zamzow |
| Dr. Nicole | Eberbach |
| Lara | Balkie |
| Tanja | Kretzschmann |
| Matthias | Gehrig |
| Matthias | Bandorf |
| Kilian | Keck |
| Dr. Jan | Allmanritter |
| Shahid | Rafique |
| Mona | Finster |
| Dr. med. Ingo | Baumgart |
| Sabine | Heumüller-Klug |
| Hans-Jürgen | Koglin |
| Prof. Dr. Olaf | Gefeller |
| Dr. Christine | Gall |
| Prof. Dr. Annette B. | Pfahlberg |
| Isabelle | Kaiser |
| Prof. Dr. Jörg | Scheidt |
| Johannes | Drescher |
| Yannic | Siebenhaar |
| Dr. Florian | Wogenstein |
| Dr. Dirk | Reinel |
| Prof. Dr. Beatrix | Weber |
| Fabian | Zarzitzky |
| Prof. Dr. Bernhard | Liebl |
| Prof. Dr. Caroline | Herr |
| Dr. Katharina | Katz |
| Prof. Dr.Dr. Andreas | Sing |
| Dr. Alexandra | Dangel |
